# Enterovirus C recombination groups: RNA sequence similarity and the viral polymerase underpin sexual replication mechanisms

**DOI:** 10.1128/jvi.00434-25

**Published:** 2025-06-24

**Authors:** Evan M. Okolovitch, Vishnu Govindarajan, Refugio Robles-Sikisaka, Grace Campagnola, Brian J. Kempf, Andrew L. Routh, Olve B. Peersen, David J. Barton

**Affiliations:** 1 Department of Immunology and Microbiology, University of Colorado Anschutz Medical Campus 129263, Aurora, Colorado, USA; 2 Department of Biochemistry and Molecular Biology, Colorado State University 548293 , Fort Collins, Colorado, USA; 3 Department of Immunology and Microbiology, Scripps Research 4356, La Jolla, California, USA; St. Jude Children's Research Hospital, Memphis, Tennessee, USA

**Keywords:** picornavirus, enterovirus, poliovirus, positive-strand RNA virus, RNA recombination, RNA-dependent RNA polymerase, RDRP

## Abstract

Enteroviruses frequently recombine with one another in nature; however, it is unclear how viral replication machinery can distinguish between related and unrelated partners during recombination. We hypothesize that viral RNA recombination involves two parental RNA templates, nascent RNA products, and their dynamic interactions with the viral polymerase—a sexual replication strategy. When nascent RNA products move from one parental RNA template to another, RNA sequence similarity may be an important factor underpinning the mechanism and efficiency of recombination. To test this hypothesis, we focused on recombination between two related group C enteroviruses, poliovirus and Coxsackievirus A21 (CVA21), using bioinformatic, biological, and biochemical approaches. Bioinformatic analyses comparing 22 prototypical group C enteroviruses delineated four recombination groups where viruses in each group exhibit high RNA sequence and amino acid similarity in their polymerase genes. ClickSeq and ViReMa methods detect recombinant forms of poliovirus with P3 genes from CVA21, analogous to recombinant circulating vaccine-derived polioviruses (cVDPV). Biochemical assays show that poliovirus and CVA21 polymerases can detect mismatched base pairs as they traverse an extended primer grip surface adjacent to the active site. Mismatched base pairs in the −2 and −3 positions destabilize polymerase elongation complexes, consistent with the predicted role of RNA sequence similarity in recombination. Two subgroup-specific genetic elements, upstream open-reading frames (uORFs) and RNase L competitive inhibitor RNAs (RNase L ciRNAs), reinforce the existence and biological relevance of enterovirus C recombination groups. Altogether, our observations suggest that enterovirus RNA replication machinery can distinguish between related and unrelated partners during recombination.

**IMPORTANCE:**

Viral RNA recombination transforms live-attenuated polioviruses into neurovirulent circulating vaccine-derived polioviruses, complicating the planned eradication of poliovirus. When humans are co-infected with poliovirus and related non-polio enteroviruses, viral replication machinery can produce recombinant viruses. However, who recombines with whom? What factors determine whether two distinct viruses can produce recombinant progeny that are fit for transmission from person to person? In this study, we clarify which viruses recombine with one another in nature and further elucidate the mechanisms by which the viral polymerase distinguishes between related and unrelated RNA templates—a sexual form of replication. Understanding these mechanisms could lead to better strategies for virus control and/or eradication.

## INTRODUCTION

Enteroviruses, like all picornaviruses, are evolutionarily ancient (1). They have small single-stranded, positive-sense RNA genomes that encode capsid proteins in the first portion of the polyprotein open-reading frame (ppORF) and non-structural proteins required for viral replication in the remainder of the ppORF (2). Many, but not all enteroviruses, encode a second upstream open-reading frame (uORF) that encodes a short upstream protein (UP) implicated in non-lytic virus egress (3). By convention, and consistent with the polyprotein processing cascade, there are three sequential regions of the ppORF: P1, P2, and P3 (4). The P1 region contains capsid proteins (VP4, VP2, VP3, and VP1), whereas the P2 (2A, 2B, and 2C) and P3 (3A, 3B, 3C, and 3D) regions contain nonstructural proteins. The P1, P2, and P3 regions of the ppORF appear to function as modular gene cassettes, wherein enteroviruses often acquire entire P2 and/or P3 regions of the genome following recombination events (5).

Following the expression of the RNA-dependent RNA polymerase (3D) and other viral proteins, viral mRNA becomes a template for replication on membrane-bound replication organelles (6, 7). Enterovirus replication occurs exclusively via RNA intermediates, wherein positive-strand RNA templates are copied into negative-strand RNA, which in turn is copied back into positive-strand RNA (8). The majority of viral RNA replication is asexual, where a single parental RNA serves as the template for replication. However, enterovirus recombination involving two parental RNAs is a sexual replication strategy that is well-established experimentally (9–12). In this case, viral RNA replication involves nascent RNA products moving from one parental RNA template to another during negative-strand RNA synthesis (12). Because RNA recombination shares ancient origins with other aspects of viral replication, one might expect that enteroviruses evolved mechanisms to specifically mediate such processes. The viral polymerase has been clearly implicated in recombination (10, 12–14); however, the precise mechanisms of enterovirus recombination are poorly understood (15, 16).

We hypothesize that viral RNA recombination, a sexual replication mechanism, involves two parental RNA templates, nascent RNA products, and their dynamic interactions with the viral polymerase (17). Asexual RNA replication, with one parental RNA template, is fast and efficient, allowing enteroviruses to produce vast amounts of progeny in short periods of time. However, asexual replication by itself renders viruses susceptible to error catastrophe (18). Sexual RNA replication with two parental RNA templates is infrequent and inefficient, producing very few progeny over long periods of time. However, sexual RNA replication is advantageous because it purges lethal mutations from viral RNA, effectively counteracting error catastrophe (19). Progeny from sexual replication can refresh the pool of viral RNAs undergoing asexual replication when a cell is infected with a single virus or lead to novel recombinant viruses when two distinct viruses co-infect a cell. Together, asexual and sexual replication strategies allow enteroviruses to thrive. However, how does the viral polymerase mediate both asexual and sexual RNA replication? In this study, we test how an extended primer grip region adjacent to the active site of the polymerase can detect RNA sequence complementarity between nascent RNA products and RNA templates.

One consequence of sexual RNA replication is recombination between related enteroviruses in humans (20, 21) and animal hosts (22). When hosts are coinfected with related viruses, nascent RNA products can move from one parental RNA to another during negative-strand RNA synthesis in coinfected cells, producing a recombinant virus. However, who recombines with whom? What factors determine whether two enteroviruses can produce recombinant progeny that is fit for transmission from host to host in nature? In this study, we use bioinformatics approaches to clarify which group C enteroviruses recombine with one another. The current investigation builds directly upon previous work in the field, especially that related to polymerase structure-function (23), recombination (17), and enterovirus subspecies groups (24).

Picornavirus taxonomy includes order, family, genus, species, and virus (25), as summarized online by The Pirbright Institute (http://picornavirales.org/). However, not all viruses within a species group are compatible partners for recombination (26). In the case of species C enteroviruses, which includes three serotypes of poliovirus (PV 1–3) and 20 other non-polio enteroviruses, it is clear that polioviruses can recombine with one another (27, 28), and with some of the non-polio enteroviruses (29, 30). Capsid genes, which define serotypes, are not amenable to recombination between serotypes (31); however, other regions of the genome, including the P2 and P3 regions, are often shared between different viruses within a species group (32). Jiang and colleagues characterized some of the factors influencing compatible partners for recombination (33), finding interactions between 2C (a P2 protein) and capsid proteins that are compatible with fitness in some contexts but incompatible in others. The poliovirus eradication campaign, with global surveillance for both wildtype and vaccine-derived viruses, provided insights into enterovirus recombination in human populations. Oral poliovirus vaccine (OPV) strains recombine with non-polio enteroviruses (NPEV) to produce recombinant viruses that transmit from person to person (30, 34, 35). These circulating vaccine-derived polioviruses (cVDPVs), which are phenotypically indistinguishable from wildtype polioviruses, cause outbreaks of poliomyelitis (36). cVDPVs contain capsid genes from one parental RNA (OPV) and portions of their genome from another parental RNA (NPEVs). A novel oral poliovirus vaccine (nOPV), which was designed to prevent reversion to virulence in the field (37), is genetically unstable due to recombination (38), leading to outbreaks of cVDPV (39, 40).

## MATERIALS AND METHODS

### Virus cDNAs, HeLa cells, co-transfections, and plaque assays

Poliovirus Type 1 Mahoney and CVA21 Kuykendall cDNAs were used to make viral RNA transcripts suitable for co-transfection into HeLa cells. Poliovirus cDNA contained a lethal GDD deletion in the active site of the polymerase (3D^pol^ ∆GDD), as previously reported (12). CVA21 cDNA (41) was kindly provided by Mathias Gromeier (Duke University). We engineered the CVA21 cDNA to contain an in-frame deletion in the capsid genes (deletion of 2,067 nucleotides between nt 945 and 3,011), creating a subgenomic CVA21 replicon analogous to that described in poliovirus (12, 42).

Poliovirus ∆GDD and CVA21 subgenomic replicon RNAs were produced from Mlu1-linearized cDNAs by T7 transcription (Ampliscribe T7 high-yield transcription kit; Cellscript Inc.). One microgram of each RNA was mixed with Transmessenger (Qiagen) and co-transfected into HeLa cells plated in 35 mm 6-well dishes, using four replicates. Following transfection, 2 mL of the culture medium (Dulbecco-modified Eagle medium containing 100 units of penicillin and 100 µg per mL of streptomycin, 10% fetal bovine serum, and 10 mM MgCl_2_) was added to each well, and the cells were incubated at 37°C in 5% CO_2_. Virus was harvested from the cells at 6 days post-transfection and amplified by two sequential passages in HeLa cells in T-150 flasks.

Plaque assays in HeLa cells were used to quantify the virus and visualize plaque phenotypes.

### Next-generation sequencing

The virus recovered from co-transfected cells was purified from 8 mL of the culture medium by centrifugation through 30% (wt/vol) sucrose cushions. Virion RNA was isolated by phenol-chloroform-isoamyl alcohol extraction, and cDNA was synthesized using ClickSeq methods (43–45). Briefly, 250 ng of the purified RNA was reversed-transcribed with SuperScript III (Invitrogen) using a random primer containing overhangs of a partial Illumina i7 adaptor and in the presence of azido-nucleotides (AzNTPs) at a ratio of 1:35 AzNTPs:dNTPs. Purified azido-blocked cDNA fragments were “click-ligated” to the Illumina i5 adaptor. Click-linked cDNA was then PCR amplified and indexed using barcoded p7 and p5 adaptors with 18 cycles of PCR. Pooled and indexed libraries were sequenced on an Element AVITI system using a Cloudbreak FS Kit yielding 2 × 150 nt paired-end reads that retain the stranded-ness of the original template.

### Bioinformatic procedures

ViReMa bioinformatic pipelines were used to map cross-over sites between poliovirus and CVA21 RNAs (46, 47). Raw demultiplexed FASTQ files were quality-filtered and adapter-trimmed using *fastp* (48) with the following parameters: -a AGATCGGAAGAGC -U --umi_loc read1 --umi_len 14 g -l 50. Trimmed and filtered FASTQ files were mapped to the virus (Poliovirus: V01149; CVA21: AF546702) and host (hg38) reference genomes using *ViReMa (v0.28*) with default settings and the following parameters: --X 3 N 2 --MicroInDel_Length 5 --Defuzz 0. *ViReMa* reports the annotations of discovered recombination events in BED6 format for intragenic recombination events and BEDPE format for intergenic recombination events, such as those between PV and CVA21. These files provide coordinates, the reference genome and strandedness of each mapped recombination event, and the number of reads that map over each specific event.

### Enterovirus RNA sequence similarity

Twenty-three prototypic group C enterovirus serotypes were selected for RNA sequence comparisons (Table 1). Viruses were listed in sub-species groups C1, C2, and C3 as reported by Brouwer and colleagues (24). NCBI reference sequences for complete genome sequences are listed for each virus, except for EV-C95, where a complete coding sequence was used (Table 1). SSE (49) and SnapGene software (https://www.snapgene.com/) were used to align viral RNA sequences. SimPlot, kindly provided by Stuart C. Ray (Johns Hopkins), was used to compare RNA sequence similarity of enterovirus RNA genomes (50). Reference strains and sliding windows of 9–300 bases, with an increment of 1 or 10, are noted in figure legends.

**TABLE 1:** Enterovirus species groups^*a*^

Species	Serotypes/genotypes
*Enterovirus* *alphacoxsackievirus* (EV Species-A)	Coxsackievirus (CV): CVA2, CVA3, CVA4, CVA5, CVA6, CVA7, CVA8, CVA10, CVA12, CVA14, and CVA16 Enterovirus (EV-): A71, A76, A89, A90, A91, A92, A114, A119, A120, A121, A122, A123, A124, and A125
*Enterovirus betacoxsackievirus* (EV Species-B)	Coxsackievirus (CV): CVB1, CVB2, CVB3, CVB4, CVB5, CVB6, and CVA9 Echovirus (E): E1 to E7, E9, E11 to E21, E24 to E27, and E29 to E31 Enterovirus (EV-): B69, B73 to B75, B77 to B88, B93, B97, B98, B100, B101, B106, B107, and B110 to B114
*Enterovirus coxsackiepol* (EV Species-C)	Polioviruses: **PV1, PV2, and PV3** Coxsackievirus: CVA1, **CVA11, CVA13, CVA17**, CVA19, **CVA20, CVA21**, CVA22, and CVA24 Enterovirus (EV-): C95, C96, **C99**, C102, C104, C105, C109, C113, C116, C117, and C118
*Enterovirus deconjuncti* (EV Species-D)	Enterovirus (EV-): D68, D70, D94, D111, and D120
*Enterovirus alpharhino* (RV Species-A)	Rhinovirus (RV-): A1, A2, A7, A8, A9, A10, A11, A12, A13, A15, A16, A18, A19, A20, A21, A22, A23, A24, A25, A28, A29, A30, A31, A32, A33, A34, A36, A38, A39, A40, A41, A43, A45, A46, A47, A49, A50, A51, A53, A54, A55, A56, A57, A58, A59, A60, A61, A62, A63, A64, A65, A66, A67, A68, A71, A73, A74, A75, A76, A77, A78, A80, A81, A82, A85, A88, A89, A90, A94, A96, A100, A101, A102, A103, A104, A105, A106, A107, A108, and A109
*Enterovirus betarhino* (RV Species-B)	Rhinovirus (RV-): B3, B4, B5, B6, B14, B17, B26, B27, B35, B37, B42, B48, B52, B69, B70, B72, B79, B83, B84, B86, B91, B92, B93, B97, B99, B100, B101, B102, B103, B104, B105, and B106
*Enterovirus cerhino* (RV Species-C)	Rhinovirus (RV-): C1 to C57

^*a*^ The *Enterovirus* genus contains 15 species groups, seven of which shown here contain common human pathogens. Polioviruses, including OPV and nOPV vaccine viruses, recombine with a subset of group C enteroviruses (in bold): Polioviruses and CVA11, CVA13, CVA17, CVA20, CVA21, and EV-C99 (29, 30, 38, 51–53).

### Polymerase expression, purification, and biochemical characterization

Poliovirus and CVA21 polymerases with solubilizing L446D mutations were expressed and purified from *E. coli* as previously described (54). Polymerases with an L420A mutation were compared with wildtype polymerases because the L420A mutation, which inhibits RNA recombination, disrupts protein-RNA interactions adjacent to the active site (12, 17, 19). Primer-template RNAs contain a 17-base long template region, a six-residue PEG linker followed by a four-base primer sequence, and a 5’ amino group labeled with IRdye 800RS NHS ester (Li-Cor Biosciences) for visualization. The six-residue (18 atom) PEG linker was used instead of a conventional RNA tetraloop hairpin structure to avoid biasing the base-pairing of the short four nucleotide priming/annealing regions. Mismatched base pairs at the N^−1^, N^−2^, and N^−3^ positions were designed into primer-template RNAs to test whether sequence similarity adjacent to the polymerase active site impacts biochemical aspects of polymerase activity. The m0 RNA has no mismatched base pairs, whereas m1, m2, and m3 RNAs each have a single mismatched A-C base pair at the N^−1^, N^−2^, and N^−3^ positions, respectively.

### Polymerase initiation and stability assays

Polymerase initiation assays were performed by incubating 5 μM 3D^pol^ with 1 μM total RNA (see below) and either 50 μM GTP to yield stalled +1 elongation complexes or 50 μM each GTP and UTP to yield stalled +2 elongation complexes, as previously described (55). The total RNA in each reaction consisted of 0.75 μM m# RNA and 0.25 μM control RNA (Ctrl) that has the same primer/template sequence but is three nucleotides shorter in the downstream template region so that it runs at a different position on the gel and can be independently quantified as an internal control in the reaction; 1 μL initiation reaction samples was quenched at various time points up to 120 min with 19 μL of quench buffer containing 50 mM EDTA, 75 mM NaCl, 50 mM HEPES pH 7.0, and 1 mM TCEP. Samples were run on denaturing 7 M urea TBE gels, imaged on an Odyssey CLx scanner, and initiation times were determined by fitting the fraction of initiated RNA vs. time to a single exponential curve. The initiation times were then divided by those observed for the internal control RNA in the same reaction, with standard RMS error propagation of the two curve-fitted initiation rate errors.

The stabilities of stalled polymerase elongation complexes were assessed by first initiating with GTP or GTP + UTP for 2.5 min at room temperature, diluting into high salt buffer to prevent further initiation or reinitiation on dissociated RNA, and then testing what fraction of the pre-initiated RNA could be further elongated with discrete samples taken over a 2 h time course; any RNA still bound in a stalled complex will be further elongated in a 30 s elongation step, whereas dissociated RNA will not rebind the polymerase under the high salt conditions. Initiated complexes were diluted 10-fold into 400 mM NaCl, 4 mM MgCl_2_, 50 mM HEPES pH 7.0, and 2 mM TCEP, and the elongation complexes were chased by adding 80 μM (final) of the next cognate nucleotide in the sequence; UTP for stalled +1 complexes and CTP for stalled +2 complexes; 5 μL reaction samples were chased with 5 μL NTP for 30 s prior to quenching with EDTA and analysis by denaturing gel electrophoresis and imaged as previously described (55). The stability of WT and L420A polymerases is assessed by plotting the fraction of elongated RNA versus time and fitting it to a decreasing single exponential curve to determine the elongation complex lifetime.

## RESULTS

### Polioviruses recombine with a subset of species C enteroviruses

The *Picornaviridae* family is taxonomically divided into five subfamilies, 68 genera, and 159 species groups (56), as summarized online by The Pirbright Institute (https://www.picornaviridae.com/). In 2024, enterovirus species groups A–D were renamed to implement a binomial nomenclature system (57, 58). Enterovirus species groups A–D are now: *Enterovirus alphacoxsackie* (EV-A), *Enterovirus betacoxsackie* (EV-B), *Enterovirus coxsackiepol* (EV-C), and *Enterovirus deconjuncti* (EV-D) (Table 1). The *Enterovirus coxsackiepol* (EV-C) species includes three poliovirus serotypes (PV 1-3), nine Coxsackievirus serotypes, and eleven enterovirus serotypes. Polioviruses recombine with some, but not all viruses listed in *species C* (EV-C) (30, 51, 52). The International Committee on Taxonomy of Viruses (ICTV) does not subdivide viruses below the rank of species; however, the biological reality of subgroups, including recombination groups, is not disputed (24, 57). As we elaborate below, polioviruses exchange genetic elements with a subset of viruses listed in *species C* (EV-C).

### Enterovirus C recombination groups

**Fig 1:**
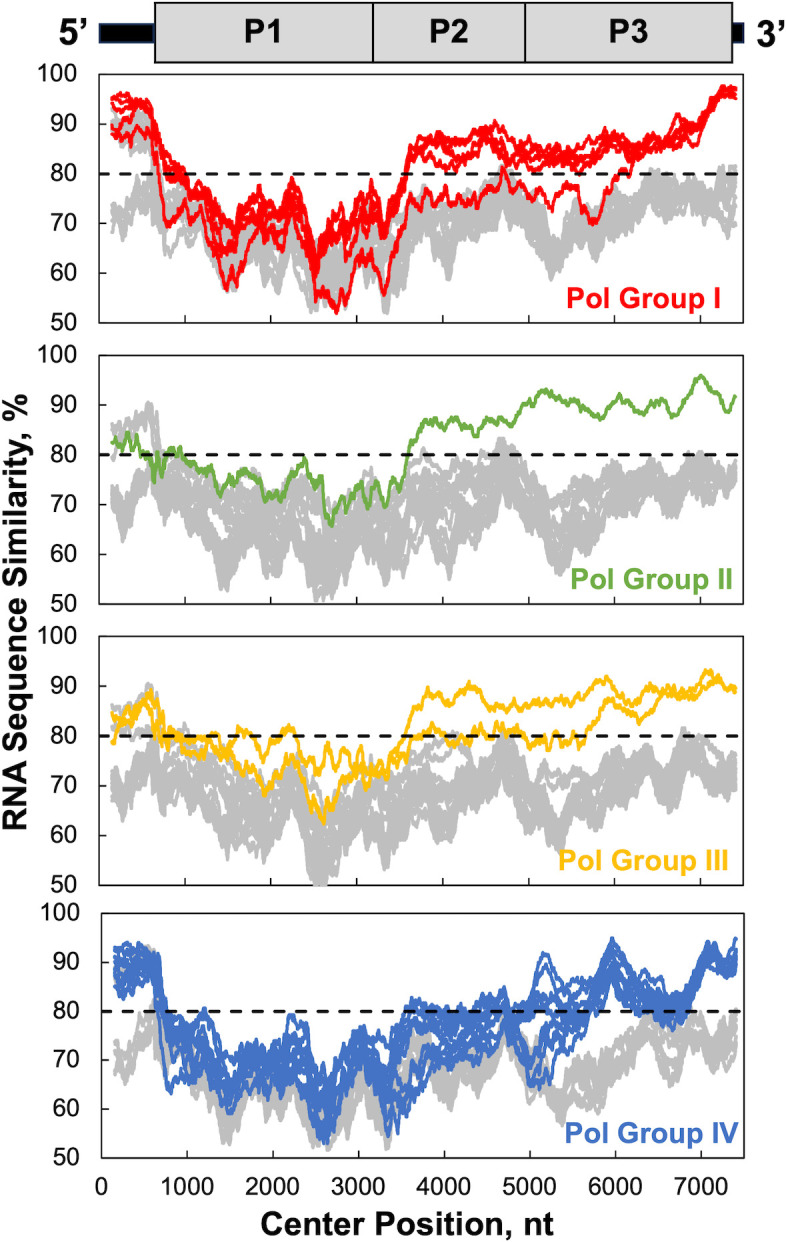
RNA sequence similarity delineates four polymerase groups/four EV-C recombination groups. SSE (49) and SimPlot (50) were used to compare RNA sequence similarity of 22 enterovirus C complete genome sequences representing each serotype (Table 2). RNA sequence similarity was compared using a 300-base sliding window with a one nt step and plotted from the 5’ to 3’ end of RNA genomes. Reference strains from each polymerase group included CVA1 (Pol Group I, red), EV-C104 (Pol Group II, green), EV-C105 (Pol Group III, yellow), and PV1 (Pol Group IV). Lines are color-coded for viruses in recombination groups I (red); II (green); III (yellow); and IV (blue) as indicated in Table 3. A colored line for the reference strain is not evident in each panel because the reference strain would have 100% sequence similarity to itself in SimPlot. As a result, Pol Group I, II, III, and IV panels have one less strain in their respective graphs. EV-C95 in Pol Group IV was not included in the analysis here due to having only a partial genome sequence. Note how well viruses in each subgroup exhibit increased RNA sequence similarity in the P2 and P3 regions of the genome when compared with viruses in other subgroups (grey lines for viruses in other subgroups for comparison in each panel).

Species C enteroviruses were initially described as a taxonomic group by Brown and colleagues at the US Centers for Disease Control and Prevention (CDC) (53). They recognized that viruses in this group shared sequence similarity in the non-capsid coding region consistent with recombination, and subsequent work in the field described three enterovirus C subspecies groups based on VP1 capsid protein sequences: C1, C2, and C3 (24, 32). For our analyses, we selected complete genome sequences from representative viruses in each subspecies group (Table 2). Simple Sequence Editor (SSE) (49) and SimPlot (50) were used to compare RNA sequence similarity of 22 complete enterovirus C genomes whose sequence similarity was plotted from 5’ to 3’ end (Fig. 1). This analysis reveals the existence of four distinct enterovirus C recombination groups when reference strains from distinct polymerase groups are used (24): CVA1 (Pol Group I), EV-C104 (Pol Group II), EV-C105 (Pol Group III), and PV1 (Pol Group IV) (Fig. 1; Table 3). High RNA sequence similarity in the polymerase gene is a cardinal feature of viruses within each recombination group (Fig. 1, note how colored lines rise above gray lines in non-capsid coding regions P2 and P3). RNA sequence similarity is the lowest in the capsid coding region (P1), where intertypic recombination between serotypes does not produce viable virus particles capable of transmission in nature. In contrast, RNA sequence similarity is higher in the non-capsid P2 and P3 coding regions, especially among other viruses within the same recombination group. RNA sequence similarity is also higher in the 5’ UTR among viruses within the same recombination group, although this region of the genome is not strictly distinct among viruses within recombination groups.

**TABLE 2:** Enterovirus C reference strains and characteristics

	Virus	Strain	NCBI	Receptor	uORF	RNase L ciRNA	3D^pol^ group	Citation
EV-C1								(24)
1	CVA1	Tompkins	AF499635	Unknown	No	No	I	(53)
2	CVA19	NIH-8663 (Dohi)	AF499641	Unknown	No	No	I	(53)
3	CVA22	Chulman	AF499643	Unknown	No	No	I	(53)
4	EV-C104	AK11	AB686524	Unknown	Yes	No	II	(59)
5	EV-C105	34S	JX514943	Unknown	Yes	No	III	(60)
6	EV-C109	NICA08-4327	GQ865517	Unknown	Yes	No	III	(61)
7	EV-C113	BBD-83	KC344834	Unknown	No	No	I	(62)
8	EV-C116	126	JX514942	Unknown	No	No	I	(60)
9	EV-C117	LIT22	JX262382	Unknown	Yes	No	II	(63)
10	EV-C118	ISR10	JX961708	Unknown	Yes	No	III	(64)
EV-C2								(24)
11	CVA21	Kuykendall	AF546702	ICAM-1	No	Yes	IV	(29)
12	CVA24	EH24/70	D90457	Sialic acid	Yes	Yes	IV	(65)
13	^*a*^EV-C95		KM273014	Unknown	Yes	Yes	IV	(32)
14	EV-C96	BAN00-10488	EF015886	Unknown	Yes	No	I	(66)
15	EV-C99	BAN00-10461	EF015008	Unknown	Yes	Yes	IV	(67)
EV-C3								(24)
16	PV1	Mahoney	V01149	CD155	Yes	Yes	IV	(53)
17	PV2	Lansing	M12197	CD155	Yes	Yes	IV	(53)
18	PV3	Leon	K01392	CD155	Yes	Yes	IV	(53)
19	CVA11	Belgium-1	AF499636	Unknown	Yes	Yes	IV	(53)
20	CVA13	Flores	AF499637	ICAM-1	Yes	Yes	IV	(53)
21	CVA17	G-12	AF499639	Unknown	Yes	Yes	IV	(53)
22	CVA20	IH-35	AF499642	ICAM-1	Yes	Yes	IV	(53)
23	EV-C102	BAN99-10424	EF555645	Unknown	Yes	Yes	IV	(67)

^*a*^ EV-C95: complete coding sequence; no complete genome.

**TABLE 3:** Enterovirus C recombination groups

Polymerase group	Enterovirus C recombination groups
I	CVA1, CVA19, CVA22, EV-C96, EV-C113, and EV-C116
II	EV-C104 and EV-C117
III	EV-C105, EV-C109, and EV-C116
IV	PV1, PV2, PV3, CVA11, CVA13, CVA17, CVA20, CVA21, CVA24, EV-C95, EV-C99, and EV-C102

### Sequence similarity in 3D polymerase genes defines recombination groups

**Fig 2:**
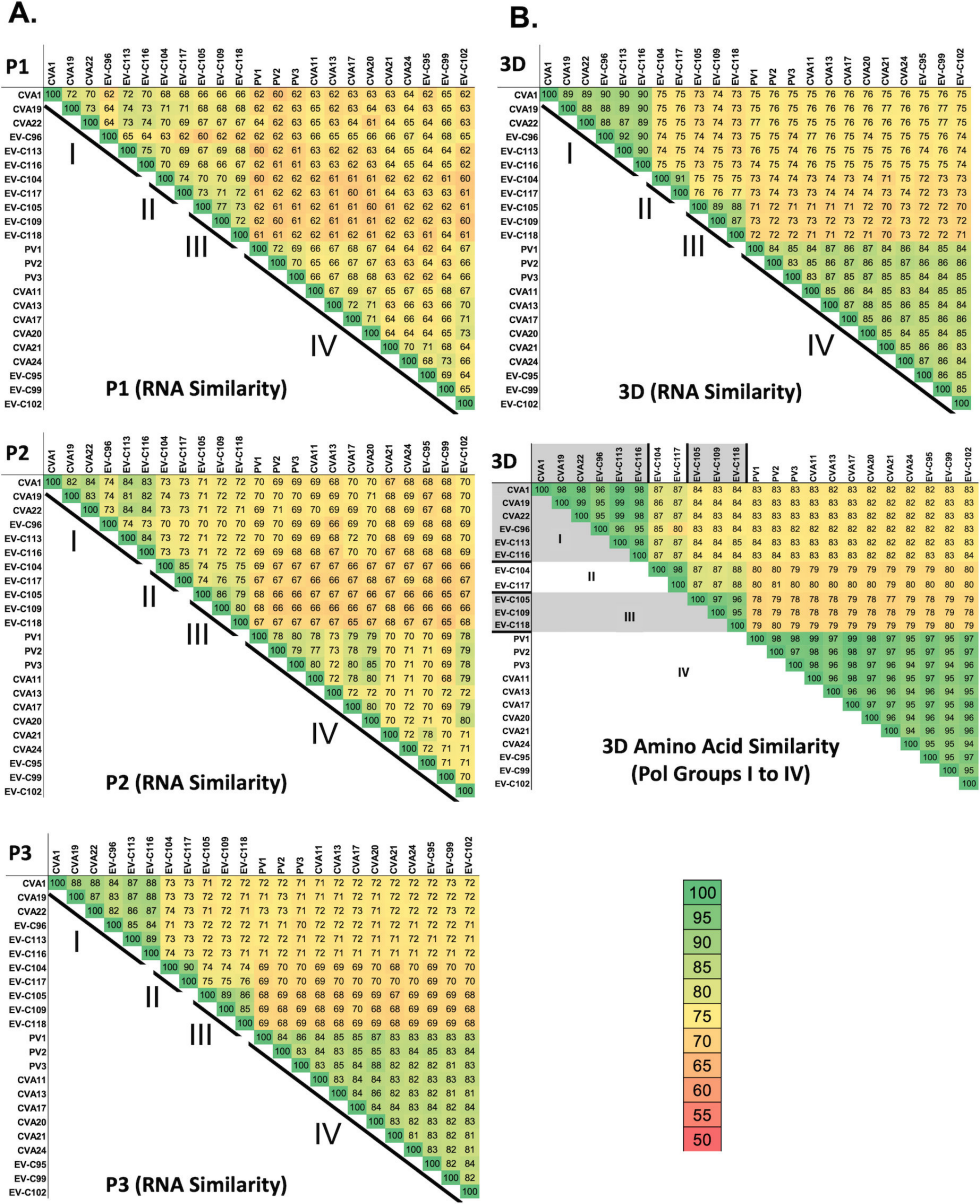
Group C enterovirus sequence similarity pairwise comparisons. (**A**) RNA sequence identity of P1, P2, and P3 regions. (**B**) RNA and amino acid sequence identity of polymerase genes. Viruses in recombination groups I, II, III, and IV are indicated by diagonal lines. Heatmap indicates amounts of sequence similarity in pairwise comparisons.

We used pairwise comparisons of group C enteroviruses to further calibrate the degree of sequence similarity in the P1, P2, and P3 regions of the genome and their 3D polymerase coding and protein sequences (Fig. 2). RNA sequence identity between individual pairs of group C enteroviruses ranged from 60% to 75% in the P1 region, 66% to 86% in the P2 region, and 68% to 90% in the P3 region. These data (Fig. 2A), like those above (Fig. 1), show that RNA sequence similarity is the lowest in the capsid coding region (P1) and highest in the P3 region, especially among viruses within each recombination group. RNA sequence similarity in the P3 region is clearly higher (> 81%) for pairs of viruses within a recombination group than for pairs of viruses in distinct recombination groups (68%–76%). Nucleotide and amino acid sequence similarity in the polymerase gene delineates most clearly which group C enteroviruses belong in each of the recombination groups; RNA sequence identity in the polymerase gene was >83% for pairs of viruses within a recombination group while amino acid identity was >94% (Fig. 2B).

Enterovirus recombination groups contain viruses that co-circulate in human populations and co-infect individual cells where they produce chimeric progeny that replicate efficiently and spread from person to person. Although these events rely on multiple factors, including cellular receptors that support co-infections of individual cells (Table 2), polymerase genes are characteristically shared among the chimeric viruses within a recombination group (Fig. 2B). Consequently, polymerase groups, based on comparisons of an individual viral gene, define the viruses present in recombination groups (Fig. 2B; Table 3).

**Fig 3:**
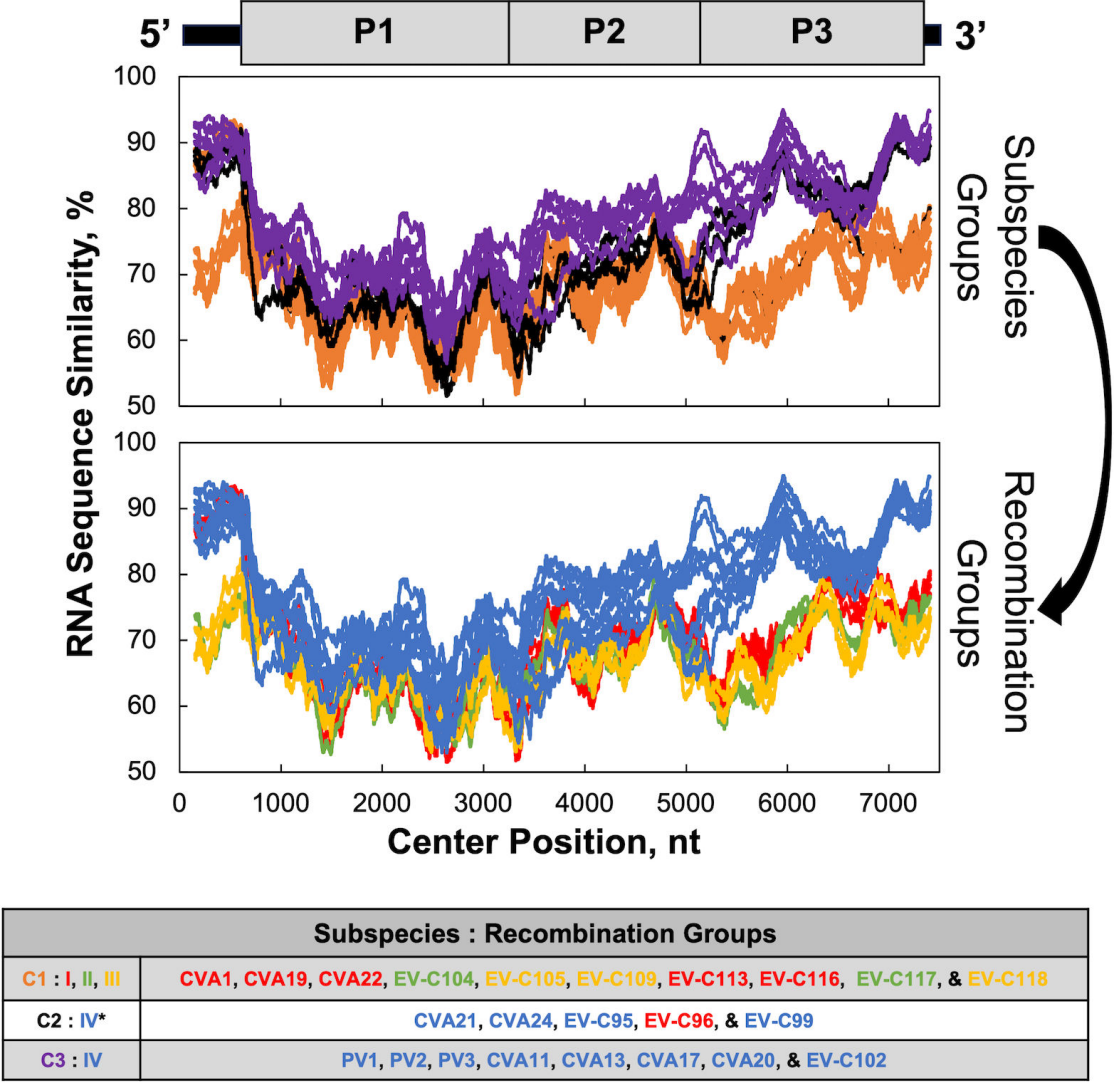
Enterovirus C subspecies groups compared with recombination groups. Enterovirus subspecies groups C1, C2, and C3 are based on capsid gene sequence similarity (24). Regrouping subspecies C1, C2, and C3 enteroviruses by polymerase and RNA sequence similarity delineates four subgroups. This new categorization emphasizes polymerase homology and recombination groups rather than capsid genes and serotypes. All the viruses in subspecies C2 and C3 are in polymerase group 4/recombination group 4, with one exception (EV-C96). Viruses in subspecies C1, along with EV-C96, are subdivided into three polymerase/recombination groups.

Figure 3 highlights the redistribution of viruses from capsid-based subspecies groups (C1, C2, and C3) into these new recombination groups. All the viruses in subspecies C2 and C3 are in polymerase group 4/recombination group 4, with one exception (EV-C96). The subspecies C1 viruses, along with EV-C96, subdivide into three of the polymerase/recombination groups. We, like others (24), conclude that recombination in nature is restricted to viruses within each recombination group. As described below, we elucidate molecular mechanisms by which enteroviral polymerases can detect RNA sequence similarity to distinguish between related and unrelated partners during recombination.

### Recombination in the field and in the lab

**Fig 4:**
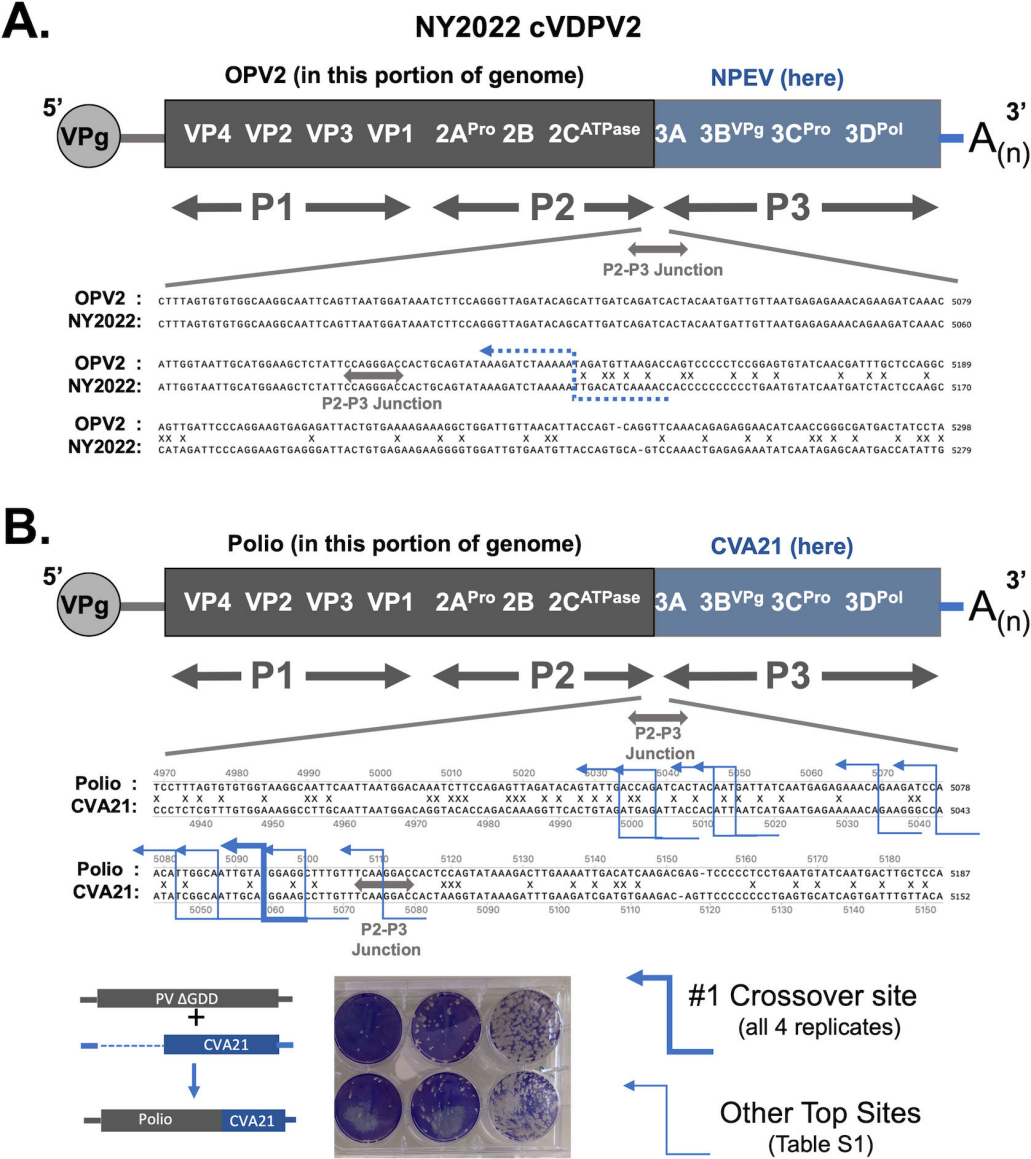
Poliovirus recombination in the field and in the lab. (**A**) cVDPV2 from 2022. Diagram of Rockland NY 2022 cVDPV2 RNA genome (OP265178). SnapGene alignment of Rockland NY 2022 (OP265178) & OPV2 (AY184220.1) reveals a crossover site adjacent to the P2-P3 junction in the viral open-reading frame. This new strain of cVDPV2 arose in 2022, has been implicated in paralytic disease, and was detected by wastewater testing in the US/Canada/London and Israel (36). Location of the crossover site is in blue. (**B**) Recombination between poliovirus and CVA21. HeLa cells were co-transfected with PV∆GDD RNA + CVA21 sgRNA (four independent replicates). Virus recovered from co-transfected cells was amplified by one passage in HeLa cells, detected by plaque assay and sequenced to detect crossover sites (Blue arrow lines). Table S1 indicates locations of the most abundant PV-CVA21 crossover sites, with >250 cDNA reads.

Recombination is readily apparent when cDNA sequences of OPV and cVDPV strains are aligned (Fig. 4A). Alignment of Rockland NY2022 cVDPV2 (OP265178) and OPV2 (AY184220.1) reveals a recombination crossover site near the P2–P3 junction in the viral open-reading frame. The two sequences are the same upstream of this crossover site, indicating OPV2 as the source of the P1 and P2 regions of the NY2022 cVDPV2 genome. Downstream from the crossover site, one sees polymorphisms between OPV2 and NY2022 (Fig. 4A, X marks locations of polymorphisms). Polymorphisms are likely tolerated because they are predominantly at wobble positions and have minimal impact on the protein sequence. These data suggest the P3 region of the NY2022 cVDPV2 genome was derived from an unknown NPEV. This new strain of cVDPV2 arose in 2022, has been implicated in paralytic disease, and was detected by wastewater testing in the US, Canada, London, and Israel (68).

Poliovirus recombination in the field (Fig. 4A) can be recapitulated in the lab (Fig. 4B). We chose to examine recombination between PV and CVA21 because PV reportedly arose from C-cluster coxsackie A virus ancestors (33), our data indicate that PV and CVA21 recombine in nature (Fig. 1 and 2; Table 3), and infectious cDNA clones are available for both viruses (41, 42). In this experiment, full-length poliovirus RNA containing a lethal mutation (∆GDD) in the viral polymerase was co-transfected with a CVA21 subgenomic RNA replicon into HeLa cells. Independently, neither RNA transcript would be capable of rescuing a viral infection. However, recombination between the two transcripts would provide a viable viral genome that could be detected using standard virological approaches. Virus recovered from co-transfected cells was amplified by one passage in HeLa cells, detected by plaque assay, and sequenced to detect crossover sites. We performed four independent replicate experiments using combinations of two separate preparations of poliovirus (∆GDD) genomic RNAs and two separate preparations of CVA21 subgenomic RNA. RNA recovered from purified virions was analyzed by next-generation sequencing (NGS) using the “ClickSeq” approach, which is a preferred method for cDNA library synthesis due to its ultralow artifactual chimera rate that would yield artefactual recombination events (44, 45). Pooled libraries were sequenced on an Element Aviti flowcell, yielding 7-8M 150 bp reads per biological replicate (Table S1A).

The output NGS data were mapped to the reference viral genomes (PV∆GDD and CVA21) using the *ViReMa* pipeline with greater than 99% of the reads from each replicate mapping to the viral genomes. Read coverage over the viral genomes for each replicate is shown in Fig. S1. Poliovirus RNA sequence was detected in the P1 and P2 portions of the genome, whereas CVA21 RNA sequence was detected in the P3 regions of the genome. *ViReMa* detects both non-homologous and homologous recombination sites both within and between the viral genomes. The frequency of these recombination sites found at the P2-P3 junction was between 87K and 111K reads (Table S1A), which correlates closely to the read coverage over each viral reference genome adjacent to these regions (Fig. S1).

Table S1B lists the locations of PV-CVA21 crossover sites: cDNA reads where poliovirus sequence was detected in one portion of the cDNA read, and CVA21 sequence was detected in the other portion of the cDNA read (Fig. 4B; Blue arrow lines). Polymorphisms between the two viral sequences pinpoint the location of crossover sites (Fig. 4B, X marks locations of polymorphisms between poliovirus and CVA21 RNA sequences). The most frequently observed crossover event, observed in all four replicates, has the coordinates PV∆GDD:5094^CVA21:5062 (Fig. 4B). Another less frequent crossover event was also observed in all four replicates (Fig. 4B; Table S1B, PV∆GDD:5087^CVA21:5053). Like NY2022 cVDPV2, crossover sites in the poliovirus recovered from this experiment were adjacent to the P2-P3 junction in the viral open-reading frame (Fig. 4B). Other minor, infrequent events were also observed in the same region but not in all four replicates, indicating that multiple possible recombination positions can yield viable progeny, but specific loci are favored (due either to positive selection of progeny arising from this event or to preferred recombination at this site). Although the NPEV partner of NY2022 is unknown due to the frequent exchange of P3 regions from one virus to another in nature, poliovirus and CVA21 are clearly the partners for genetic exchange in this experimental setting. These data, and data from others (33), show that non-polio enteroviruses from Pol Group IV/Recombination Group IV can form chimeric progeny with poliovirus, consistent with our bioinformatic analyses (Fig. 1 and 2; Table 3).

**Fig 5:**
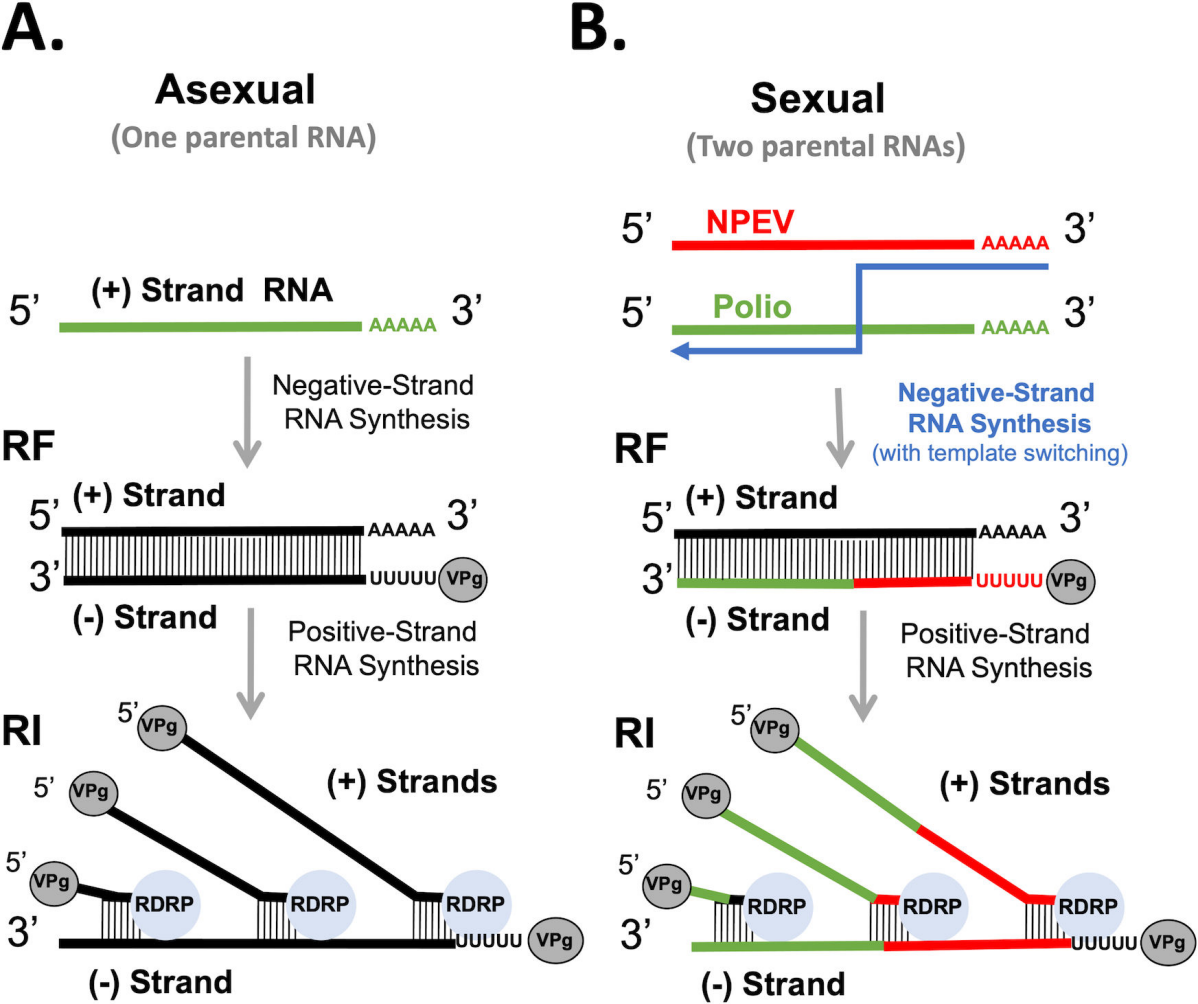
Diagram of asexual and sexual RNA replication strategies. (**A**) Asexual RNA replication with one parental template. Replicative form (RF) and replicative intermediate (RI) RNAs. (**B**) Sexual RNA replication is the same as asexual RNA replication, except there are two parental templates (e.g., Polio & NPEV) with template-switching during negative-strand RNA synthesis. Two parental templates are copied into one chimeric (-) strand, which is then copied into multiple (+) strand progeny.

Diagrams of asexual and sexual RNA replication provide a framework to test and understand enterovirus recombination (Fig. 5). Asexual RNA replication, with one parental RNA template, involves negative-strand RNA synthesis followed by positive-strand RNA synthesis (Fig. 5A). One copy of negative-strand RNA is made from one positive-strand RNA template, producing a dsRNA replicative form (RF). Multiple copies of positive-strand RNA products are made from each negative-strand RNA template, producing a replicative intermediate (RI). Sexual RNA replication is the same as asexual RNA replication, except there are two parental templates (e.g., Polio and NPEV) with template-switching during negative-strand RNA synthesis (Fig. 5B). Two parental templates are combined into one chimeric negative strand, which is then copied into multiple positive-strand progeny and subject to selection.

### Enterovirus polymerases detect RNA sequence similarity adjacent to the active site

**Fig 6:**
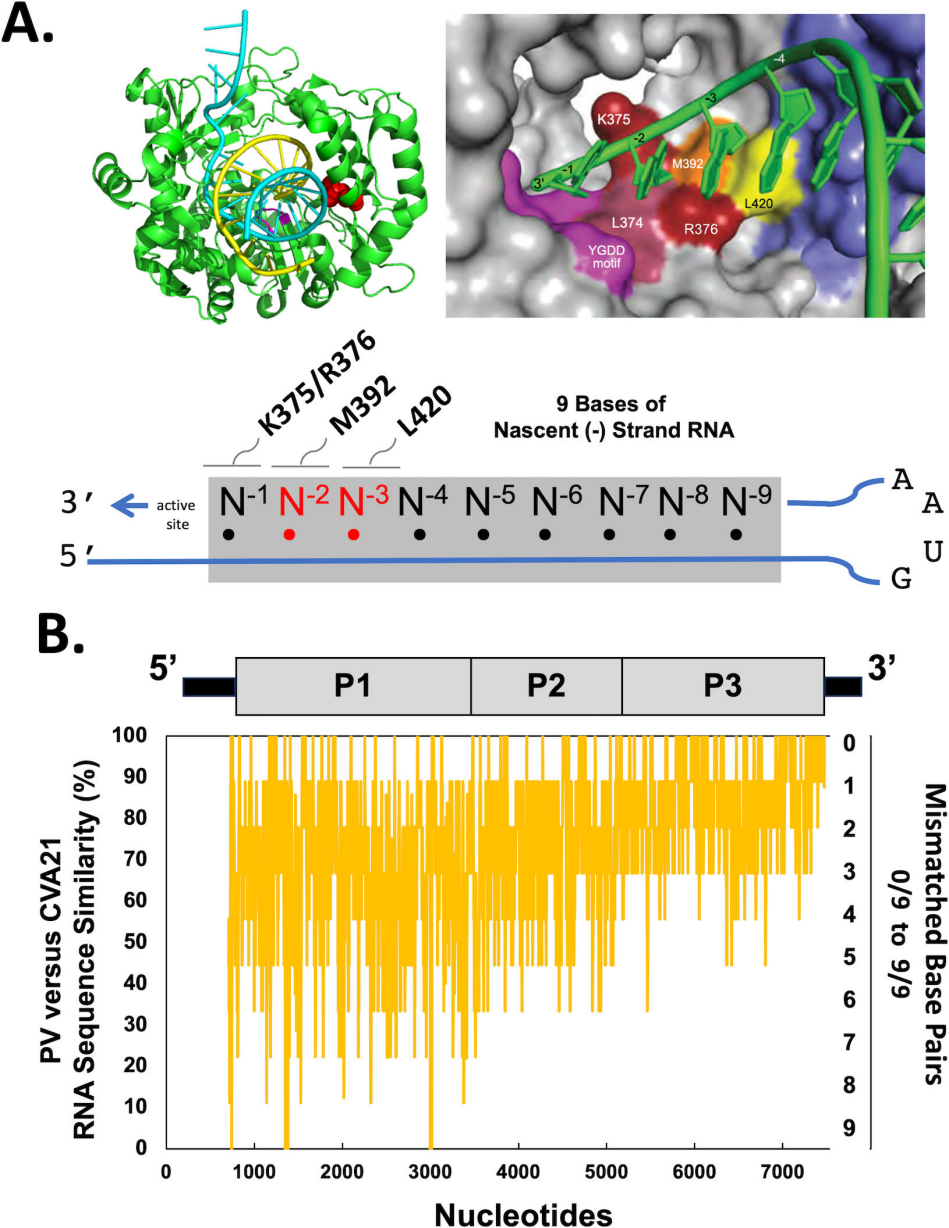
Enterovirus polymerases detect nascent RNA products and sequence similarity adjacent to the active site. (**A**) Polymerase elongation complex highlighting extended primer grip residues [K375/R376 (red), M392 (orange), L420 (yellow)] interacting with nascent RNA products adjacent to the active site [YGDD motif (fuchsia)]. Diagram shows nine bases of nascent RNA (N^−1^ to N^−9^) in dsRNA products adjacent to the active site. (**B**) RNA sequence similarity of CVA21 and PV1 using a nine-base sliding window and a 1-base step across the viral ORFs. RNA sequence similarity (y-axis on the left) ranges from 0% to 100% for each 9-base window across the ORF. Mismatched base pairs (y-axis on the right) range from 0/9 to 9/9 for each 9-base window across the ORF.

When nascent RNA products move from one parental RNA template to another, RNA sequence similarity may be an important factor underpinning the mechanism and efficiency of recombination. To test this hypothesis, we considered how RNA sequence similarity can be detected by the polymerase (Fig. 6). Atomic structures show seven base pairs of dsRNA exiting the active site in polymerase elongation complexes, and an extended primer grip region composed of K375, R376, M392, and L420 interacts with the three bases of nascent RNA adjacent to the active site (Fig. 6A) (69, 70). The L420 residue interacts with the product strand N^−3^ ribose of nascent RNA products, and previous studies have demonstrated that an L420A mutation specifically disables recombination without impairing asexual replication (12, 14). Based on these features of the polymerase elongation complex, we used a 9-base sliding window to compare RNA sequence similarity between poliovirus and CVA21 ORFs (Fig. 6B). RNA sequence similarity ranges from 0% to 100% for each 9-base sliding window across the ORF, with higher sequence similarity in the P3 region and lower sequence similarity in the P1 and P2 regions. From the perspective of the polymerase and nascent RNA moving from one template to another, there are 0–3 mismatched base pairs in each sliding window across the P3 region of the ORF, 1-4 mismatched base pairs in most sliding windows across the P2 region, and 1-6 mismatched base pairs in most sliding windows across the P1 region. These data highlight how the polymerase might detect different degrees of RNA sequence similarity across the ORFs of recombining partners.

**Fig 7:**
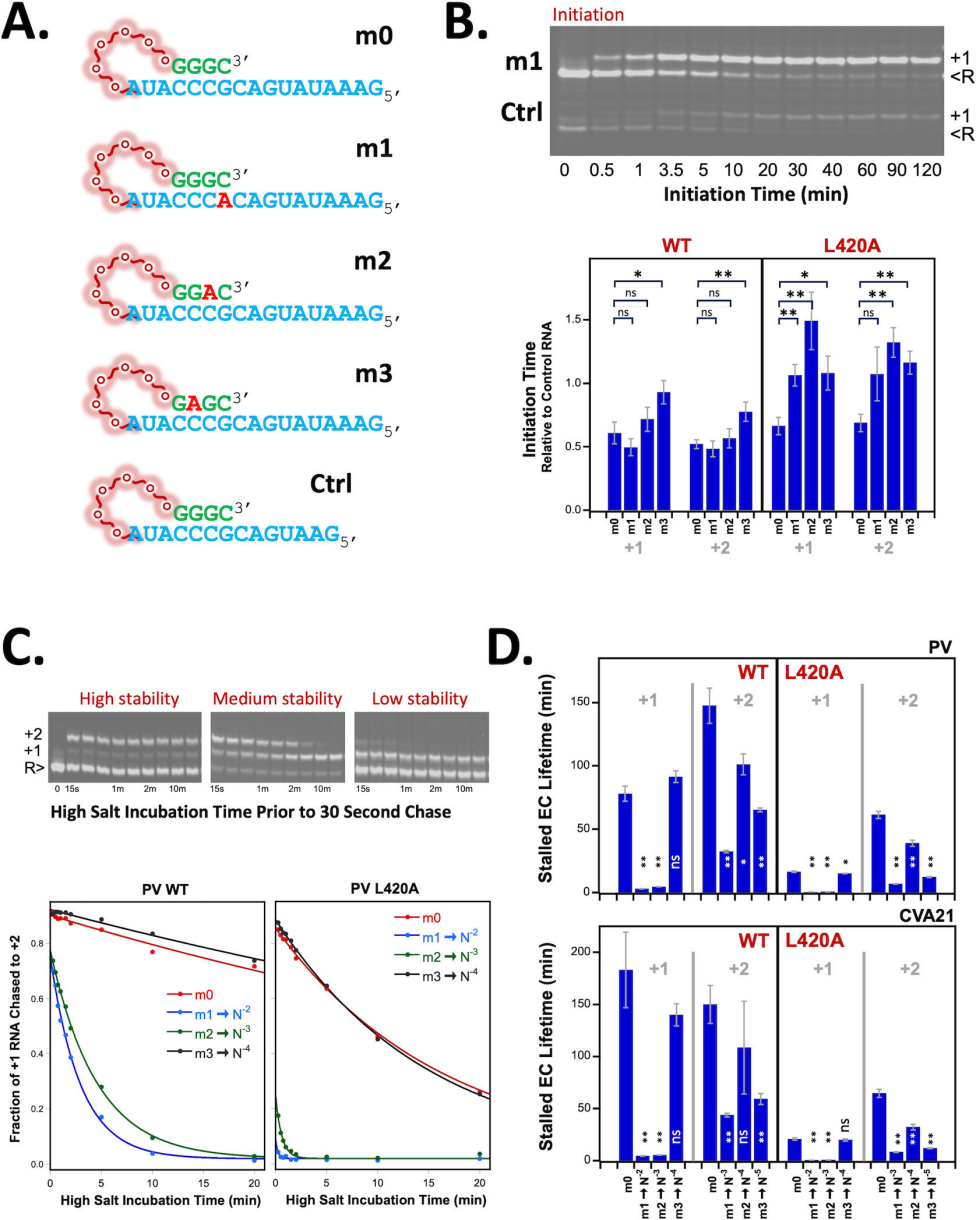
Mismatched template-primer design and *in vitro* polymerase assays. (**A**) Template-primer pairs tethered by a polyethylene glycol (PEG) linker to minimize bias for short duplex formation. 17-base template (in blue); 6-residue PEG linker (in red); 4-base primer to mimic nascent RNA (in green). m0 RNA has four base-pairs with zero mismatched, whereas m1, m2, and m3 RNAs incorporate a single mismatched base at the first (N^−1^), second (N^−2^), and third (N^−3^) positions, respectively. Ctrl RNA is the same as m0, but with a three nt deletion to separate it on gels. These template-primer pairs were designed to characterize mismatched base pairs across the extended primer grip of the polymerase. (**B**) Polymerase initiation assay with 5 μM polymerase, 0.75 μM m# RNA, 0.25 μM Ctrl RNA, and 100 μM GTP incubated from 0 to 120 min, showing +1 products from both precursor RNAs (R). Bar chart shows +1 and +2 initiation times for all m# RNAs on CVA21 wildtype and L420A polymerases, expressed relative to the initiation time for the internal Ctrl RNA in each individual reaction. *P*-values for comparisons with m0 in each data subgroup are indicated with * <0.05, ** <0.01, and ns being not significant. (**C**) Elongation complex stability experiment in which pre-initiated +1 or +2 complexes were diluted into high-salt buffer to prevent RNA rebinding and then periodically assayed to see what fraction of the complexes remain intact and can rapidly chase the +1 RNA to a + 2 product. Plots with single exponential curve fits show selective EC destabilization when mismatches are translocated into the N^−2^ and N^−3^ sites but not the N^−4^ site, and the L420A polymerase mutation exacerbates these effects by additionally destabilizing all complexes. Overall, the stability of a N^−2^ or N^−3^ mismatched RNA complex with L420A poliovirus 3D^pol^ is destabilized ~150 fold compared to a no-mismatch RNA with the wildtype polymerase. (**D**) Summary of elongation complex stability data for all four m# RNAs with both +1 and +2 initiation reactions on both wildtype and L420A mutants of PV and CVA21 polymerases. Exact values and fold effects are listed in Table 4 and *P*-values relative to m0 data are indicated as in panel B.

With these concepts in mind, we designed template-primer pairs with mismatched base pairs at the N^−1^, N^−2^, and N^−3^ position and characterized their behavior in assays with wildtype and L420A polymerases from both PV1 and CVA21 (Fig. 7). Template-primer RNAs contain a 17-base long template, a six-residue PEG linker, and a short four-base primer to mimic nascent RNA products during recombination where a long priming duplex may or may not be present (Fig. 7A). The m0 RNA has no mismatched base pairs, whereas m1, m2, and m3 have one A-C mismatched base pair at the N^−1^, N^−2^, and N^−3^ positions, respectively (Fig. 7A). A shorter control template-primer (Ctrl) was co-incubated with the m# template-primer pairs in every reaction at 3-fold lower concentration as an internal control for fully base-paired initiation efficiency. In an initiation assay containing GTP, one can see how the m1 and Ctrl RNA precursors are converted into +1 RNA products over time (Fig. 7B). A comprehensive initiation data set comparing CVA21 wildtype and L420A polymerases shows efficient initiation for both +1 and +2 reactions on all four templates, and overall, they initiated in about half the time of the internal control RNA, a difference we attribute to less-efficient RNA binding by the shorter template present at 3-fold lower concentration (Fig. 7B). For the wildtype polymerases, there are minor differences in initiation times of the different RNAs, although the m3 mismatch is somewhat slower in both reactions. In contrast, with the L420A polymerase, there is a marked ~2-fold increase in the initiation times for all three mismatched primers compared with the fully base-paired m0 RNA. These data show that single mismatches in even a short 4-nt priming duplex can slow but do not abolish elongation by 3D^pol^ and that the L420A recombination-deficient polymerase is more sensitive to mismatches than the wildtype enzyme.

**Fig 8:**
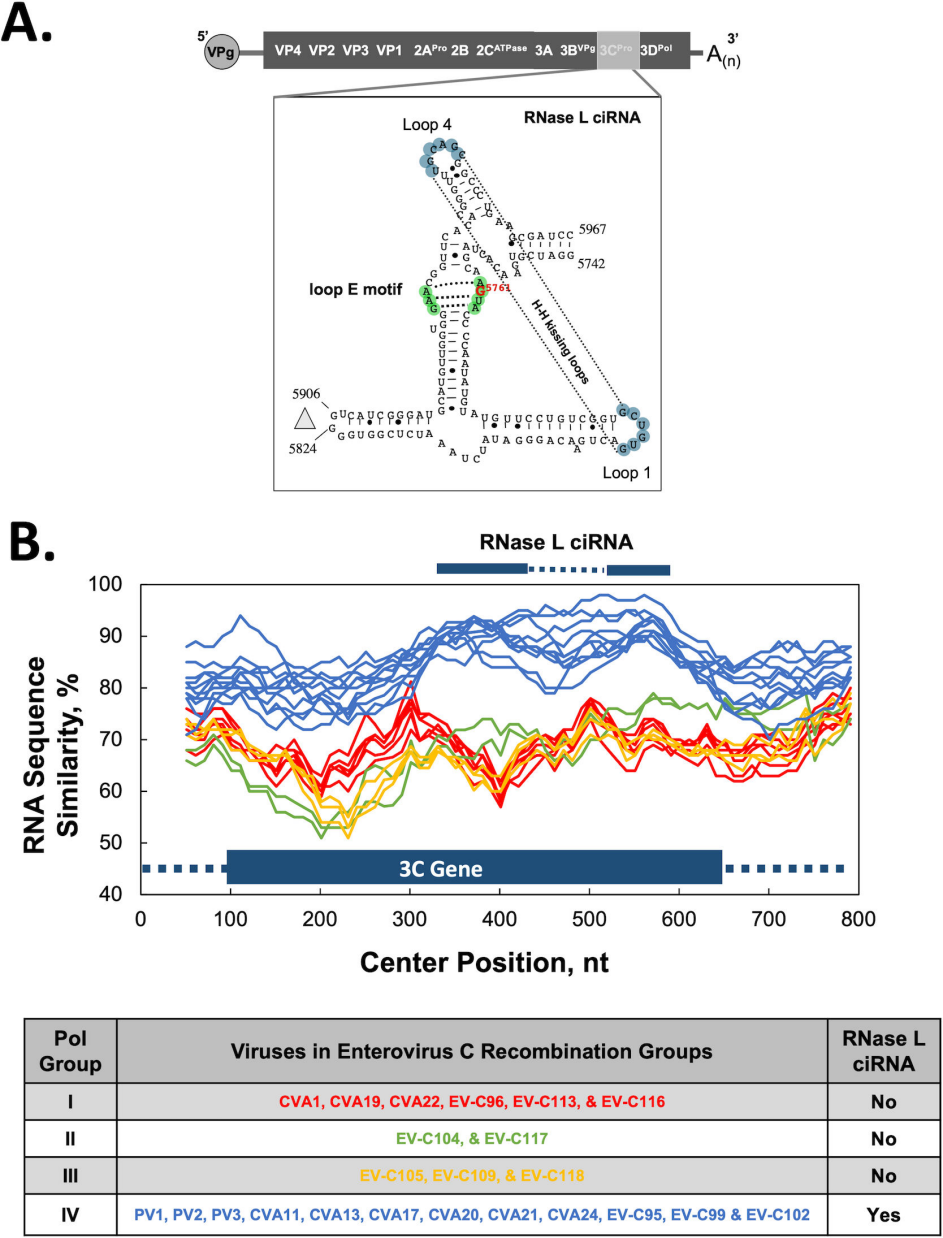
RNase L ciRNA is conserved in recombination group IV. (**A**) RNase L ciRNA, an RNA structure in the 3C ORF (71, 72). (**B**) RNA sequence similarity of the 3C gene using PV1 reference for SimPlot (100-base sliding window with 10-base step) and the representative viruses from polymerase groups I (red), II (green), III (yellow), and IV (blue). Location of RNase L ciRNA highlighting 5’ and 3’ portions (solid blue bars) and intervening sequence (dashed line). RNase L ciRNA sequence alignments (Fig. S2) show loop E motif polymorphisms incompatible with functional activity in Pol Group I, II, and III viruses.

We next examined how mismatches affect the temporal stability of stalled 3D^pol^ elongation complexes by doing a brief 2.5 min initiation reaction, diluting the sample into high salt to prevent rebinding of any dissociated RNA, and then periodically measuring how much elongation complex remained by measuring what fraction of the pre-initiated RNAs could be rapidly chased to a longer product (Fig. 7C). The initiation reactions involve one or two nucleotide incorporation and translocation events, and these shift the location of the initial mismatches in the m1-m3 RNAs to different product locations during the stability time course. Thus, a +1 initiation with GTP of the m1 RNA places the mismatch into the N^−2^ position, whereas a +2 initiation with GTP and UTP places the same mismatch into the N^−3^ position. As summarized in Fig. 8D, the 3D^pol^ elongation complex lifetime is very sensitive to mismatches at the N^−2^ and N^−3^ positions of the product duplex, but much less so at the N^−4^ position. The wildtype CVA21 elongation complex with the fully base-paired m0 RNA is stable for ~3 h, but this is reduced to ~5 min when mismatches are present at the N^−2^ and N^−3^ positions, and then reduced even further to ~30 s when the polymerase has the L420A mutation (Fig. 7D, bottom panel). These effects are seen for both PV and CVA21 wildtype polymerases that are destabilized ~20-fold and ~40-fold, respectively, when there are N^−2^ and N^−3^ mismatches compared with the non-mismatched m0 RNA (Table 4). The L420A mutation exacerbates these effects and increases sensitivity to RNA mismatches by both destabilizing a perfectly base-paired m0 RNA by 2-fold to 10-fold compared with wildtype, depending on polymerase and initiation length, and then further destabilizing the N^−2^ and N^−3^ mismatches by ≈2-fold compared with m0 (Table 4). Combining these effects, the lifetime of the L420A elongation complex with an N^−2^ or N^−3^ mismatch is reduced by ≈170-fold for PV and up to ≈400-fold for CVA21 relative to a perfect duplex on the wildtype enzymes (Table 4). Similar outcome trends, albeit with lower magnitude effects, were observed when the m1-m3 mutations were introduced into a conventional 8-base pair hairpin duplex substrate (Fig. S2). Altogether, these data show that the extended primer grip can detect RNA sequence similarity (and mismatches) adjacent to the active site of the polymerase via elongation complex temporal stability effects, allowing it to distinguish between related and unrelated partners during recombination by destabilizing nascent 3D^pol^ elongation complexes containing mismatched template-primer sequences.

**TABLE 4:** Effects of RNA mismatches and L420A mutation on elongation complex stability^*a*^

	EC stability (minutes)	∆ stability (fold vs m0 RNA)	Cumulative ∆stability (Fold vs m0 on WT 3D^pol^)
CVA21 3D^pol^			
A21 WT +1 m0	180 ± 40	1.0 ± 0.3	
m1➜N^−2^	**4.6 ± 0.2**	**40 ± 8** ^**^	**40 ± 8** ^**^
m2➜N^−3^	**5.2 ± 0.1**	**35 ± 7** ^**^	**35 ± 7** ^**^
m3➜N^−4^	140 ± 11	1.3 ± 0.3 ^ns^	
A21 WT +2 m0	150 ± 20	1.0 ± 0.2	
m1➜N^−3^	**44 ± 2**	**3.4 ± 0.4** ^**^	**3.4 ± 0.4** ^**^
m2➜N^−4^	110 ± 40	1.4 ± 0.6 ^ns^	
m3➜N^−5^	59 ± 5	2.5 ± 0.4 ^**^	
A21 420 + 1 m0	20.8 ± 1.0	1.0 ± 0.1	
m1➜N^−2^	**0.42 ± 0.10**	**50 ± 14** ^**^	**440 ± 150** ^**^
m2➜N^−3^	**0.79 ± 0.04**	**26 ± 2** ^**^	**230 ± 50** ^**^
m3➜N^−4^	19.9 ± 1.0	1.0 ± 0.1 ^ns^	
A21 420 + 2 m0	65 ± 4	1.0 ± 0.1	
m1➜N^−3^	**8.3 ± 0.4**	**7.8 ± 0.6** ^**^	**18 ± 2** ^**^
m2➜N^−4^	32 ± 3	2.0 ± 0.2 ^**^	4.7 ± 0.7 ^**^

PV 3D^pol^			
PV WT + 1 m0	78 ± 6	1.0 ± 0.1	
m1➜N^−2^	**3.0 ± 0.1**	**26 ± 2** ^**^	**26 ± 2** ^**^
m2➜N^−3^	**4.5 ± 0.1**	**17 ± 2** ^**^	**17 ± 2** ^**^
m3➜N^−4^	91 ± 5	0.9 ± 0.1 ^ns^	
PV WT + 2 m0	148 ± 14	1.0 ± 0.1	
m1➜N^−3^	**32 ± 1**	**4.6 ± 0.5** ^**^	**4.6 ± 0.5** ^**^
m2➜N^−4^	101 ± 8	1.5 ± 0.2 ^*^	
m3➜N^−5^	65 ± 2	2.3 ± 0.2 ^**^	
PV 420 + 1 m0	16.5 ± 0.5	1.0 ± 0.1	
m1➜N^−2^	**0.23 ± 0.11**	**72 ± 36** ^**^	**340 ± 170** ^**^
m2➜N^−3^	**0.60 ± 0.10**	**28 ± 5** ^**^	**130 ± 20** ^**^
m3➜N^−4^	15.1 ± 0.1	1.1 ± 0.1 ^*^	
PV 420 + 2 m0	61 ± 3	1.0 ± 0.1 ^ns^	
m1➜N^−3^	**6.9 ± 0.2**	**8.9 ± 0.5** ^**^	**21 ± 2** ^**^
m2➜N^−4^	39 ± 2	1.6 ± 0.1 ^**^	3.8 ± 0.4 ^**^
m3➜N^−5^	12.3 ± 0.4	5.0 ± 0.3 ^**^	

^*a*^ EC Stability ± StdErr based on curve fitting the chase data to a single exponential. The second column lists the fold-effect destabilization arising from mismatches compared with the m0 RNA in the same +1 or +2 initiation condition, and the third column shows the cumulative effect relative to the wildtype enzyme with m0 RNA under the same +1 or +2 initiation condition, with RMS error propagation. * and ** indicate *P*-values < 0.05 and <0.01, respectively. Bold text highlights the strong effects arising when the mismatched bases are translocated into the N^−2^ and N^−3^ positions.

## DISCUSSION

Enterovirus recombination groups contain viruses that exchange genetic material with one another in nature. The exchange of genetic material between viruses in each recombination group occurs in the non-capsid coding regions where RNA sequence similarity between viruses is the highest (Fig. 1 and 2). RNA sequence similarity between serotypes is the lowest in the P1 capsid coding region, where intertypic recombination between serotypes does not produce viable virus capable of transmission in nature. RNA and amino acid sequence identity in the polymerase gene of group C enteroviruses delineate four recombination groups (Fig. 1 and 2), where recombination in nature is restricted to viruses within each recombination group (Table 3). Periodic genetic exchange in the non-capsid coding regions maintains recombination groups over time.

### RNA sequence similarity and the viral polymerase underpin mechanisms of recombination

The highest RNA sequence similarity between members of recombination groups is evident in the polymerase gene (Fig. 2B). This is interesting considering the location of the polymerase gene in viral RNA and the role of the polymerase protein in the mechanisms of recombination. Recombination occurs when nascent RNA products move from one parental RNA template to another during negative-strand RNA synthesis (Fig. 5). Because the polymerase gene is present at the 3’ end of the viral RNA genome, nascent negative-strand RNA products will invariably contain complementary RNA sequence from the polymerase gene of the first parental RNA. When these nascent negative-strand RNA products move to another parental RNA template, RNA sequence complementarity between nascent negative-strand RNA products and positive-strand RNA templates will guide realignment with homologous RNA sequences. Thereafter, a polymerase must engage the nascent negative-strand RNA products and resume elongation on the second parental RNA template. As we have previously described (17), an extended primer grip in the viral polymerase interacts directly with three bases of nascent negative-strand RNA products on the second parental RNA template (Fig. 6). Remarkably, this region of the viral polymerase can detect RNA sequence similarity in this context, as mismatched base pairs in the 2nd and 3rd positions of nascent RNA products destabilized polymerase elongation complexes (Fig. 7). Furthermore, an L420A polymerase mutation in the extended primer grip further destabilizes polymerase elongation complexes, exacerbating the degree of destabilization caused by mismatched base pairs at the 2nd and 3rd positions of nascent RNA products (Table 4). These data reveal mechanisms by which an RNA organism (enteroviruses) can distinguish between related and unrelated partners during RNA replication and recombination. Furthermore, these mechanisms highlight why RNA sequence similarity in the polymerase gene (Fig. 2B) is a cardinal feature of viruses within each recombination group. According to these mechanisms, enterovirus RNA recombination sites are not randomly selected across host and viral RNAs, as suggested by others (15). Rather, RNA sequence complementarity between nascent negative-strand RNA products and positive-strand RNA templates can guide and favor realignment at homologous RNA sequences in viral genomes.

Because the extended primer grip of enterovirus RDRPs is structurally and functionally conserved across enterovirus and rhinovirus species groups (17, 23, 54, 69, 73), the mechanisms outlined here are broadly applicable. We expect that recombination (sub)groups across all enterovirus and rhinovirus species groups can be more clearly defined by (re)applying the bioinformatic approaches (Fig. 1 and 2) and theoretical principles (Fig. 5 and 6) highlighted herein. 3D polymerase amino acid sequence similarity of 94% or more is reliable evidence for recombination between viruses in nature (Fig. 2B) (20, 21, 26, 31, 53, 74). Notably, these ideas and theoretical principles first arose in the field long ago (9, 29).

### EV-C subgroup-specific genetic elements

Subgroup-specific genetic elements reinforce the existence and biological relevance of enterovirus C recombination groups. The first genetic element, RNase L ciRNA, is an RNA structure in the 3C ORF (71, 72, 75) present exclusively in viruses from polymerase group IV/EV-C recombination group IV (Fig. 8). The RNase L ciRNA contains two key features conserved in these viruses: an H-H kissing loop and a loop E motif (Fig. 8A). Mutations in and around the loop E motif disable the RNA’s ability to inhibit RNase L (75). RNA sequence similarity suggests that the RNase L ciRNA is conserved in viruses in polymerase group IV, but not in viruses in polymerase groups I, II, or III (Fig. 8B). Conserved loop E motifs present in RNA sequence alignments suggest that polymerase group IV viruses contain a functional RNase L ciRNA (Fig. S3). In contrast, viruses in polymerase groups I, II, and III contain loop E polymorphisms incompatible with functional RNase L ciRNA activity (Fig. S3). The presence of a functional RNase L ciRNA in polymerase group IV viruses, but not other viruses, suggests that RNase L antiviral activity may be more intense in one or another of the host tissues where these viruses replicate. However, in many cases, the cellular receptors for group C enteroviruses are unknown (Table 2), and the selective nature of host cells in the gut and respiratory tract is largely undefined.

The second genetic element, uORFs, is distinct from one enterovirus C polymerase group to another (Fig. S4). uORFs begin at an AUG codon in domain VI of the IRES and encode 42–73 residue long Upstream Proteins (UPs) via frameshifted overlap with the beginning of the ppORF (3). uORFs and corresponding UPs are found in Pol Group II, III, and IV viruses; however, the UPs encoded therein are distinct from one polymerase group to another. uORFs are absent in Pol Group I viruses, with the exception of EV-C96 whose uORF resembles those in polymerase group IV, highlighting the (unique) ability of this virus to recombine with viruses in two recombination groups (polymerase groups I and IV), as reported by others (24). Premature termination codons (*PTC) and indels disrupt the expression of some UPs; however, even in those cases, the gene segments remain largely intact from one recombination group to another (Fig. S4; Table S2). Because uORFs and UPs are largely uncharacterized, one can only speculate why they are expressed in some viruses and not in others (3). If UPs mediate aspects of non-lytic virus egress exclusively in gut epithelial cells, they might contribute to prolonged virus replication in such tissues, yet be dispensable when viruses replicate in other tissues, such as the respiratory tract. These possibilities require further investigation.

### Taxonomy and recombination groups

We (data herein), and others (24), provide evidence of four enterovirus C recombination groups, where recombination in nature is restricted to viruses within each recombination group. Regrouping enteroviruses by their ability to recombine with one another (recombination groups) is akin to classic definitions of biological species in eukaryotes where sexual replication mechanisms underpin species groups (76). As we highlight, recombination with two parental RNAs is a sexual strategy of enterovirus replication (Fig. 5). The subgroup-specific distribution of two genetic elements, uORFs and RNase L ciRNAs, reinforces the existence and biological relevance of enterovirus C recombination groups. However, the taxonomic classification of viruses by the ICTV is not based on sexual strategies of replication. Rather, structural proteins and serotypes are emphasized for species-level taxonomy of picornaviruses (20, 53, 77). Picornavirus taxonomy includes order, family, genus, species, and virus (25, 56); however, not all viruses within a taxonomic species group are compatible partners for recombination (26). The ICTV does not subdivide viruses below the rank of species; however, the biological reality of subgroups, including recombination groups, is not disputed (20, 24, 26, 31, 53, 57, 74). Despite recognized flaws in the ICTV species rank for RNA viruses (78), both ICTV taxonomy and enterovirus C recombination groups can coexist as useful categorization systems for people in the field. In regard to viral species, Van Regenmortel artfully addressed the topic in his article entitled “*Viruses are real, virus species are manmade, taxonomic constructions*” (79). With this article in mind, we suggest that enterovirus recombination groups are real.

### Transmission, tropism, coinfection, and recombination

Transmission and replication of enteroviruses at mucosal surfaces of the respiratory and enteric tracts enhance opportunities for co-infections and recombination. It is well established that viruses in recombination group IV replicate in the respiratory and enteric tracts. Cellular receptors for most viruses in recombination group IV are known (Table 2) (65, 80–82), consistent with their ability to infect and replicate in common tissue culture cells that express ICAM-1 and CD155. In contrast, cellular receptors for viruses in recombination groups I, II, and III are unknown (Table 2), and many of these viruses do not infect and replicate in common tissue culture cells (62). Consequently, it is not surprising that viruses in recombination groups I, II, and III rarely, if ever, recombine with viruses in recombination group IV. Enteroviruses discovered more recently have less information regarding their tissue tropism and pathogenesis; however, they exhibit clear evidence of recombination (59–64, 66). Subgroup-specific genetic elements (Fig. 8; Fig. S2 and S3), for example, upstream open-reading frames (uORFs) and RNase L competitive inhibitor RNAs (RNase L ciRNAs), reinforce the existence and biological relevance of enterovirus C recombination groups. The proximal location of RNase L ciRNA in the 3C gene, next to the 3D polymerase gene, suggests genetic linkage with pol group IV polymerase genes during recombination (Fig. 8; Fig. S2 and S3). Genetic linkage of 3C, 3D, and RNase L ciRNAs in pol group IV viruses is intriguing considering the roles of 3CD precursor proteins in RNA replication (83), 3D polymerase in making dsRNAs that provoke innate immune pathways (84), and RNase L ciRNA in counteracting a dsRNA-activated antiviral pathway (71, 72, 75). Lulla and colleagues suggest that the uORF and UPs of viruses in recombination group IV facilitate nonlytic virus egress in gut epithelial cells (3). Furthermore, they report that rhinoviruses and respiratory tropic enteroviruses do not have uORFs and therefore do not express UPs (3). Except for EV-C96, viruses in the recombination group I do not have uORFs. EV-C96 is unique, as it appears to recombine with viruses in two recombination groups (polymerase groups I and IV) (24, 85), with an uORF and UP protein like those in polymerase group IV (Fig. S3). EV-C96 has been isolated at different times and places from stool (32, 67, 85–87), like other viruses within recombination groups I and IV. Except for EV-C96, the subgroup-specific distribution of uORFs and RNase L ciRNAs suggests that viruses in distinct recombination groups likely exhibit distinct cellular tropism in the respiratory and/or enteric tracts. A phylodynamic study of uORFs could provide clues as it relates to tissue tropism, recombination, and pathogenesis.

### Consequences of enterovirus recombination

Recombination counteracts error catastrophe (19), enhances pathogenesis (88), and provides enteroviruses access to a global gene pool that is continuously optimized and refreshed (26). This global gene pool allows viruses to replace unfit genome segments with newly optimized gene segments shared among wildtype viruses in human populations. Outbreaks of enterovirus disease typically involve new recombinant forms of individual serotypes (52, 89, 90). Oral poliovirus vaccine viruses, which, by design, contain attenuating unfit genome segments, swap their unfit genome segments with optimized gene segments as they spread from person to person (27, 35, 38, 52). This leads to cVDPVs that are phenotypically wildtype, complicating the planned eradication of poliovirus (36, 91, 92).

### Summary

RNA sequence similarity delineates four enterovirus C recombination groups, where recombination in nature is restricted to viruses within each group. Mechanistically, we show that the extended primer grip of the viral polymerase can detect RNA sequence similarity (and mismatches) adjacent to the active site of the polymerase to distinguish between related and unrelated partners during recombination. Understanding the molecular basis of enterovirus recombination groups could lead to novel approaches for virus control and/or eradication.

## Data Availability

ViReMa and associated test data are available as previously described at https://sourceforge.net/projects/virema/. Raw sequence data (FASTQ) are available on the NCBI Small Read Archive (SRA) with project number PRJNA1222474.

## SUPPLEMENTAL MATERIAL

BEDPE 1 jvi.00434-25-s0001.txt PVdeltaGDD_1-CVA21_subg1 virus fusions.

BEDPE 2 jvi.00434-25-s0002.txt PVdeltaGDD_1-CVA21_subg2 virus fusions.

BEDPE 3 jvi.00434-25-s0003.txt PVdeltaGDD_2-CVA21_subg1 virus fusions.

BEDPE 4 jvi.00434-25-s0004.txt PVdeltaGDD_2-CVA21_subg2 virus fusions.

Supplemental figures and tables jvi.00434-25-s0005.pdf Figures S1 to S4; Tables S1 and S2.

ASM does not own the copyrights to Supplemental Material that may be linked to, or accessed through, an article. The authors have granted ASM a non-exclusive, world-wide license to publish the Supplemental Material files. Please contact the corresponding author directly for reuse.
