# Supplementary material for: Enterovirus C recombination groups: RNA sequence similarity and the viral polymerase underpin sexual replication mechanisms
Source: J Virol. 2025 Jun 24;99(7):e00434-25. doi: 10.1128/jvi.00434-25 (PMC12282145; doi:10.1128/jvi.00434-25)
Supplement: Supplemental figures and tables — Figures S1 to S4; Tables S1 and S2. [file jvi.00434-25-s0005.pdf]

Supplemental Materials

Okolovitch et al. JVI 2025

Supplemental files listing details of recombination sites between PV-CVA21:

1. PVΔGDD1+CVA21sg1VirusFusions.BEDPE
2. PVΔGDD1+CVA21sg2VirusFusions.BEDPE
3. PVΔGDD2+CVA21sg1VirusFusions.BEDPE
4. PVΔGDD2+CVA21sg2VirusFusions.BEDPE

See individual files online for data from each replicate.

Table S1A. NGS Data

Sample	Total Reads	PV Mapped	CVA21 Mapped	PV-CVA21 Recombination Reads
I. PVΔGDD1 + CVA21sg1	7,421,105	3,709,995	3,581,422	87,799
II. PVΔGDD1 + CVA21sg2	7,339,783	3,587,258	3,632,394	102,511
III. PVΔGDD2 + CVA21sg1	8,143,303	3,889,032	4,093,590	97,557
IV. PVΔGDD2 + CVA21sg2	7,548,532	3,716,131	3,694,246	111,763

Table S1B. PV-CVA21 Crossover Sites*

Replicate	Top** Sites	PV-CVA21 Crossover (nt)	cDNA Reads	Top Site in all replicates
I	1	5095-5061	84719	✓
	2	5087-5053	1288	✓
	3	5110-5076	264	
II	1	5095-5061	65827	✓
	2	5046-5012	25051	
	3	5087-5053	8618	✓
	4	5081-5047	1119	
III	1	5095-5061	41848	✓
	2	5077-5043	28012	
	3	5038-5004	12137	
	4	5069-5035	6803	
	5	5046-5012	4513	
	6	5033-4999	1631	
	7	5087-5053	605	✓
IV	1	5095-5061	81078	✓
	2	5049-5015	16627	
	3	5087-5053	10740	✓
	4	5081-5047	1279	

* PVΔGDD x CVA21 sgRNA co-transfections

** PV-CVA21 Crossover sites with >250 cDNA reads.

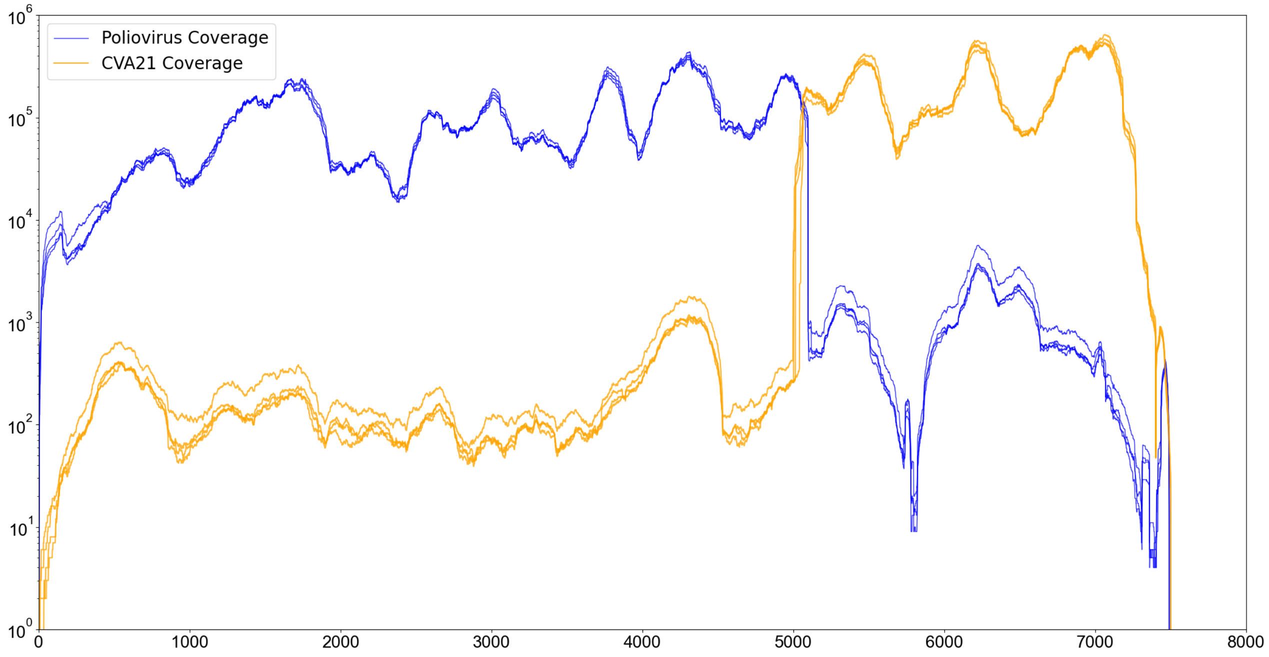

Fig. S1 Read coverage for PV and CVA21 genomes for each replicate in Table S1A. ClickSeq next-generation sequencing and Virus Recombination Mapper (ViReMa) were used to detect crossover sites between poliovirus and coxsackievirus A21 (CVA21), as shown in Table S1A, Table S1B and Figure S1 (1-4).

Supplemental files listing details of recombination sites between PV-CVA21:

1. PVΔGDD1+CVA21sg1VirusFusions.BEDPE
2. PVΔGDD1+CVA21sg2VirusFusions.BEDPE
3. PVΔGDD2+CVA21sg1VirusFusions.BEDPE
4. PVΔGDD2+CVA21sg2VirusFusions.BEDPE

See individual files online for data from each replicate.

Comparison with 8bp Duplex RNA

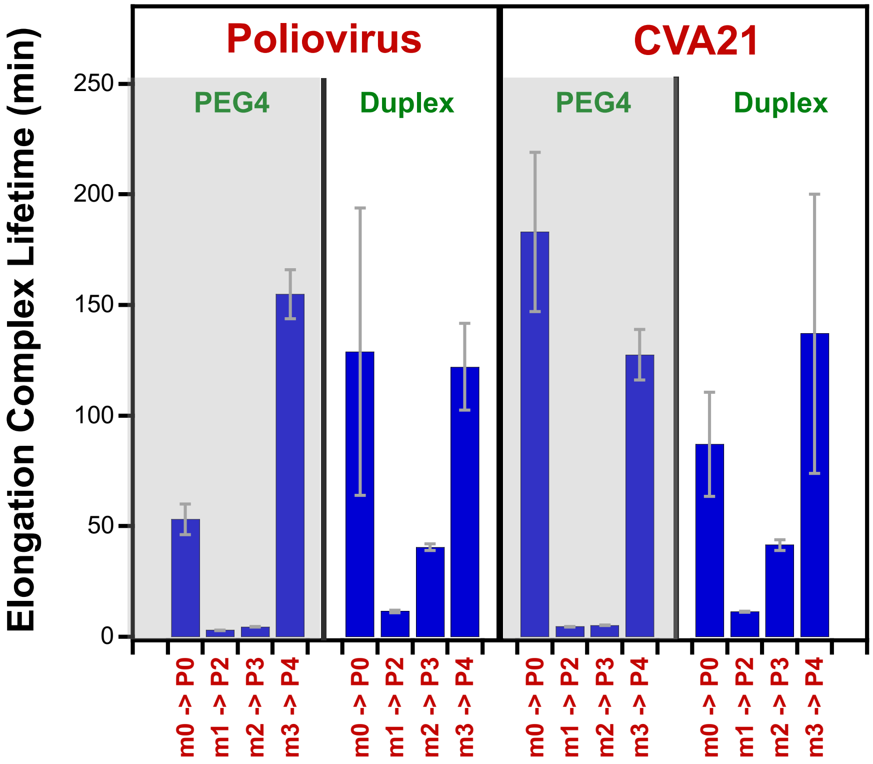

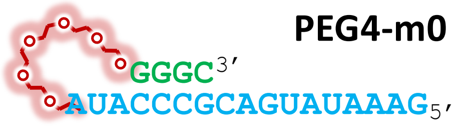

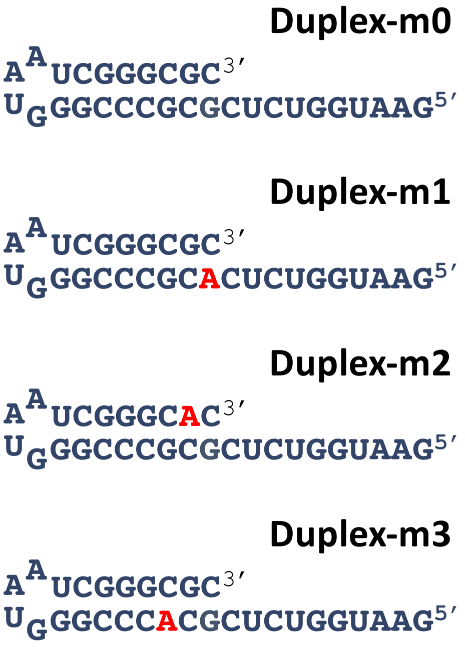

Figure S2. Mismatches at N^{-2} and N^{-3} positions also destabilize an 8 base-pair hairpin RNA, but the effects are not as large as with the shorter base-pairing region of the PEG4 RNAs. Polymerases were expressed, purified and assayed as previously reported (5-6).

A.

Pol Group IV — 5′ loop E

Virus	Sequence	Ref.
PV1	^{5742}TGATCGTGAACACTAGCA**AGTA**CCCCAATATGTATGTTCCTGTCGGTGCTGTGACTGAACAGGGATATCTAAATCTCGGTG^{5822}	*
PV2	^{5741}TAATTGTGAACACTAGTA**AGTA**CCCCAACATGTATGTTCCTGTCGGTGCTGTGACTGAACAGGGGTATCTCAATCTCAGTG^{5821}	*
PV3	^{5733}TGATTGTGAACACTAGTA**AGTA**CCCCAACATGTATGTTCCTGTCGGTGCTGTGACTGAGCAGGGATACCTAAATCTCGGTG^{5813}	*
CVA11	^{5756}TGATCGTGAACACTAGTA**AGTA**TCCCAACATGTATGTGCCTGTTGGTGCTGTGACCGAACAGGGATATCTTAATCTCGGTG^{5835}	*
CVA13	^{5760}TGATCGTGAACACTAGTA**AGTA**CCCCAACATGTATGTCCCTGTTGGTGCTGTGACCGAGCAGGGATATCTGAATCTCGGTG^{5840}	*
CVA17	^{5759}TGATCGTGAACACTAGTA**AGTA**CCCCAATATGTATGTTCCAGTTGGTGCTGTGACCGAACAGGGATATCTCAATCTTGGCG^{5839}	*
CVA20	^{5738}TGATCGTGAACACTAGTA**AGTA**CCCCAACATGTATGTTCCTGTTGGCGCTGTGACTGAACAGGGATATCTTAATCTCGGTG^{5818}	*
CVA21	^{5707}TGATCGTGAACACTAGCA**AGTA**CCCCAATATGTATGTCCCCGTTGGTGCTGTGACCGAACAGGGATATCTTAATCTCAGTG^{5787}	*
CVA24	^{5765}TGATCGTGAACACTAGTA**AGTA**CCCCAACATGTATGTTCCTGTAGGTGCTGTGACCGAACAGGGATATCTCAATCTCGGTG^{5845}	*
EV-C95	^{5679}TGATCGTGAACACTAGTA**AGTA**CCCTAACATGTATGTTCCTGTTGGTGCTGTGACCGAACAGGGATACCTCAATCTCGGTG^{5822}	**
EV-C99	^{5752}TGATCGTGAACACTAGCA**AGTA**CCCTAATATGTATATCCCTGTTGGTGCTGTAACTGAACAGGGGTACTTGAATCTCGGTG^{5832}	**
EV-C102	^{5739}TGATCGTGAACACTAGTA**AGTA**CCCCAATATGTATGTACCTGTCGGTGCAGTGACCGAACAAGGGTACTTGAACTTGGGCG^{5819}	**

Pol Group I

Virus	Sequence	Ref.
CVA1	^{5696}TGGCTGTCAACACCTCCA**AATT**TCCAAATATGTACATTCCAGTTGGATCAGTAGTTGAACAGGGAATGTTGAACCTTGGTG^{5776}	*
CVA19	^{5709}TTGCTGTAAACACCTCCA**AATT**TCCAAACATGTATATCCCAGTTGGATCAGTTGTTGAACAGGGGATGTTAAATTTAGGGG^{5789}	*
CVA22	^{5706}TGGCTGTTAACACCTCTA**AATT**CCCAAACATGTATATACCAGTTGGTTCAGTAGTTGAACAAGGAATGTTAAATCTTGGCG^{5786}	*
EV-C96	^{5776}TAGCAGTAAACACTAGTA**AATT**CCCAAACATGTACATCCCAGTTGGATCTGTCACCGAACAAGGCATGCTCAACCTTGCAG^{5856}	**
EV-C113	^{5677}TTGCTGTAAACACCTCTA**AATT**TCCAAATATGTACATACCAGTTGGGTCAGTTGTTGAACAGGGTATGTTAAATCTCGGAG^{5757}	**
EV-C116	^{5683}TTGCTGTGAACACCTCCA**AGTT**TCCAAACATGTACATTCCAGTTGGATCAGTTGTTGAGCAAGGCATGCTGAATCTTGGAG^{5763}	**

Pol Group II

Virus	Sequence	Ref.
EV-C104	^{5708}TGGCCGTTAACACCAGTA**AGTA**CCCCAACATGTTCATTCCTGTTGGTGCGGTGTCAGAGCAGGGAATGCTAAACCTTGGTG^{5788}	**
EV-C117	^{5663}TAGCTGTTAACACCAGTA**AATA**CCCCAACATGTTTATTCCCGTTGGCGCGGTGTCGGAACAGGGGATGCTAAACCTTGGTG^{5743}	**

Pol Group III

Virus	Sequence	Ref.
EV-C105	^{5625}TAGCAGTGAACACCACAC**GGTT**CCCGAACATGTATATCCCAGTGGGAGCTGTCACTGAGCAAGGAATGCTAAATTTGGGGG^{5705}	**
EV-C109	^{5654}TGGCAGTAAACACCACGC**GGTT**CCCAAACATGTACATCCCAGTTGGAGCTGTCGCTGAGCAAGGGATGCTAAACCTGGGGG^{5734}	**
EV-C118	^{5657}TAGCAGTGAACACCACGC**GTTT**CCCTAATATGTACATCCCAGTGGGAGCTGTCACTGAGCAGGGGATGCTGAACTTAGGGG^{5737}	**

* RNA Biol. 2008
** This report

B.

Pol Group IV — 3′ loop E

Virus	Sequence	Ref.
PV1	^{5906}GTCATCGGGATGCATGTTGGTGG**GAA**CGGTTCACACGGGTTTGCAGCGGCCCTGAAGCGATCA^{5968}	*
PV2	^{5905}GTCATCGGGATGCATGTTGGTGG**GAA**CGGTTCACATGGGTTCGCAGCAGCCCTGAAGCGATCC^{5967}	*
PV3	^{5897}GTCATCGGGATGCACGTTGGTGG**GAA**TGGTTCACATGGGTTTGCAGCGGCCCTGAAGCGGTCA^{5959}	*
CVA11	^{5919}GTCATCGGGATGCATGTTGGTGG**GAA**TGGTTCACATGGGTTCGCAGCGGCCCTAAAGCGATCG^{5981}	*
CVA13	^{5924}GTCATCGGGATGCATGTTGGCGG**GAA**CGGTTCACATGGGTTTGCAGCAGCCCTAAAGCGATCA^{5986}	*
CVA17	^{5923}GTGATTGGGATGCATGTTGGAGG**GAA**CGGTTCACACGGGTTCGCAGCAGCCCTGAAACGATCA^{5985}	*
CVA20	^{5902}GTCATCGGGATGCATGTTGGTGG**GAA**CGGTTCACACGGGTTTGCAGCGGCCCTGAAGCGATCG^{5964}	*
CVA21	^{5871}GTCATTGGGATGCATGTTGGCGG**GAA**CGGTTCACATGGGTTTGCAGCAGCCCTCAAGCGATCA^{5933}	*
CVA24	^{5929}GTTATTGGGATGCATGTTGGAGG**GAA**CGGTTCACATGGGTTCGCAGCAGCCCTGAAGCGGTCC^{5991}	*
EV-C95	^{5843}GTCATCGGGATGCATGTTGGAGG**GAA**CGGTTCGCATGGGTTTGCAGCAGCCCTGAAACGGTCA^{5905}	**
EV-C99	^{5916}GTTATCGGGATACATGTTGGAGG**GAA**CGGTTCACACGGGTTTGCAGCGGCCCTAAAGCGGTCA^{5978}	**
EV-C102	^{5903}GTCATTGGGATGCATGTTGGTGG**GAA**CGGTTCACATGGGTTCGCTGCGGCCCTGAAGCGGTCA^{5965}	**

Pol Group I

Virus	Sequence	Ref.
CVA1	^{5860}GTGATTGGCATACATGTTGGTGG**TAA**TGGATCTCACGGTTTCGCAGCTGCACTTAAAAGAAGT^{5922}	*
CVA19	^{5873}GTAATTGGCATACACGTTGGTGG**TAA**CGGGTCCCACGGTTTCGCTGCCGCACTTAAAAGAAGT^{5935}	*
CVA22	^{5870}GTGATTGGCATACATGTTGGTGG**CAA**TGGATCTCATGGATTTGCAGCTGCACTAAAGAGGAGC^{5932}	*
EV-C96	^{5940}GTTATTGGTATACATGTTGGAGG**TAA**TGGCAGTCATGGCTTTGCCGCAGCACTCAAAAGATCA^{6002}	**
EV-C113	^{5841}GTGATAGGCATACACGTCGGCGG**TAA**TGGATCCCATGGCTTCGCCGCTGCTCTCAAAAGAAGC^{5903}	**
EV-C116	^{5847}GTAATTGGCATACACGTTGGTGG**TAA**TGGATCCCATGGTTTTGCTGCTGCTCTTAAAAGAAGT^{5909}	**

Pol Group II

Virus	Sequence	Ref.
EV-C104	^{5872}GTGATTGGAATACACGTGGGTGG**CAA**CGGATCTCACGGCTTTGCTGCGGCCCTGAAACGGTCC^{5934}	**
EV-C117	^{5827}GTGATTGGGATACATGTGGGTGG**CAA**TGGGTCACATGGCTTTGCTGCGGCCCTGAAACGGTCC^{5889}	**

Pol Group III

Virus	Sequence	Ref.
EV-C105	^{5789}GTCATTGGTATTCACGTGGGGGG**TAA**TGGCTCCCATGGGTTCGCCGCTGCACTGAAAAGGTCA^{5851}	**
EV-C109	^{5818}GTGATTGGCATACACGTGGGGGG**TAA**TGGCTCCCATGGGTTCGCCGCTGCACTGAAAAGATCG^{5880}	**
EV-C118	^{5821}GTTATTGGCATTCATGTGGGAGG**TAA**TGGTTCCCATGGCTTTGCTGCAGCATTGAAGAGGTCA^{5883}	**

Figure S3. RNase L ciRNA sequence alignments. Conserved loop E motifs suggest Pol Group IV viruses contain a functional ciRNA. Viruses in Pol Groups I, II, and III contain loop E polymorphisms incompatible with functional RNase L ciRNA activity (7-9).

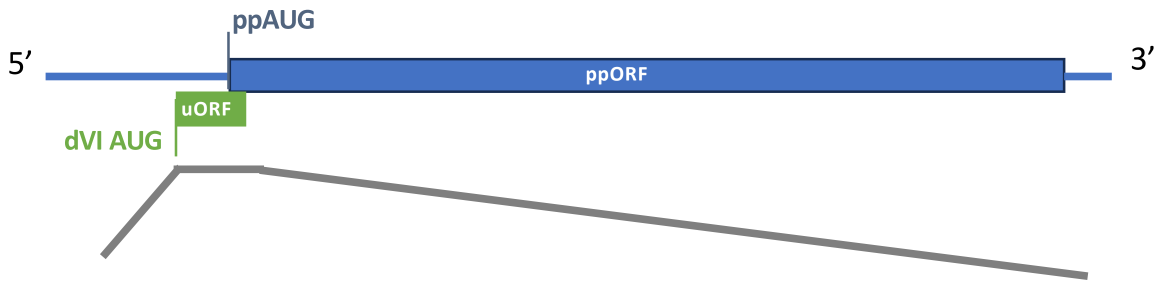


```
                1        10        20        30        40        50        60
    CVA1     MVTT*DCCHIAFGLAIR*-----------------------------------------------     *PTCΔ
    CVA19    MVTI*DCYHIAFGLAIR*-----------------------------------------------     *PTCΔ
I   CVA22    MVTI*DCYHIAFGLAIRLLFRAFHLCFVCLTRSI*------------------------------     *PTCΔ
    EV-C96   MVTILDCYHKASWIGHPVKVKVTIYLSVGFYPKTTHLPDLIRIIILLGNYYHNGCPSFITKGWCT*
    EV-C113  MVTIKDCYHIAFGLAIR*FLRL*------------------------------------------     *PTCΔ
    EV-C116  MVTI*DCYHIAFGLAIR*-----------------------------------------------     *PTCΔ

II  EV-C104  MVTIVLISCCHCSRSVCNYKNKTISCYHPPRSVHNHKNEIFCHNGRSSICSEQWNPRKQKHSHWWFNHQLHYY*
    EV-C117  MVTFIVFKCYHILRKHIVIIQSYYCRNHGCSGFSSEQWYT*

    EV-C105  MVTIV*FSCYHLPHSST*CQTD*---------------------------------        *PTC & Indels
III EV-C109  MVTIVVYSCYHSLHSEINLTLSNYLLQWVLKFPRRITVLMKIPIVRVVDQLSITPQ*
    EV-C118  MVTIVEYSCYHYCILQIVNKLRNNITQWELKCQPNTTALTRIPTVLVKGPPLITPP*

    PV1      MVTITDCYHKANWIGHPVKVRLIIYLFAGSAPLSVFTLSTISTVISIRQLYHNGCSGFITESGRT*
    PV2      MVTIIDCYHKANWIGHPVRI*LLNYSLVGIAPLKSCALTPIGITSLLRYHHHYGRPSLITESWSP*     *PTC
    PV3      MVTIIDCYHKASWIGHPV*IRLITPLFVGSTPETFYSLTY*NCLKTGFQCHNGSSSIIPKSRRSR      *PTC
    CVA11    MVTIIDCYHKASWIGHPVKVKYIIYLFVGFIPLNVHLLNLIRVTLLVRSHYHNGSSSIISESGCP*
    CVA13    MVTIIDCYHKANWIGHPVKVRLIIYLFVGFTPLNTFTPSIISIVIRIRQLYHNGCTSFITKGWSP*
IV  CVA17    MVTIIDCYHKANWIGHPVRIRSIIYLFIGFTPLTTYTYDLIRVNLLLRHLHHNGSSSFLTKSRCP*
    CVA20    MVTIIDCYHKANWIGHPVKARLTIYLLVGLTTLNTFILGVTCIAIRIKQLHHNGCASFITESGCTREH*
    CVA21    MVTIYKLLPYSYWIGHPVLCNIFECFFHKPY*---------------------------------     *PTCΔ
    CVA24    MVTIIDCYHRATWIGHPVRVRSIVYLFVGFTSITPYTPTILRIVLLIRQHHHNGSPGVFTKSWSP*
    EV-C95   MVTIIDCYHKATWIGHPVKYKHIVYLFVGFTPLTQSTLSIINIVSLIRYYYYNGCPSLNPENWCTRESKYSG*
    EV-C99   MVTIIDCYHKANWIGHPVRIKSLIYLFVGFTKINYRTLALIRIVLLIRFHHHNGSSSFVSKGWCT*
    EV-C102  MVTIIDCYHKANWIGHPVKYKHIIYLFVGFTPLTQLTPKIIFIVLLVRRHYYNGSTSIISEGGRS*
                1        10        20        30        40        50        60
```

UP Proteins

Pol Group	Viruses in Enterovirus C Recombination Groups	uORF	RNase L ciRNA
I	CVA1, CVA19, CVA22, EV-C96, EV-C113 & EV-C116	No*	No
II	EV-C104 & EV-C117	Yes	No
III	EV-C105, EV-C109, & EV-C118	Yes	No
IV	PV1, PV2, PV3, CVA11, CVA13, CVA17, CVA20, CVA21, CVA24, EV-C99 & EV-C102	Yes	Yes

* EV-C96 has an uORF, an exception among viruses in Pol Group I.

Figure S4. uORFs and the predicted proteins encoded therein. uORFs and UP proteins are distinct from one enterovirus C polymerase group to another. Diagram shows how uORFs begin at an AUG codon in domain VI of the IRES and overlap with the ppORF (10). uORFs and corresponding UP proteins are absent in Pol Group I viruses (but for one exception, EV-C96). uORFs are found in Pol Group II, III and IV viruses; however, the UP proteins encoded therein are distinct from one polymerase group to another. Premature termination codons (*PTC), deletions (--) and indels disrupt expression of some UP proteins. For uORF characteristics, see Table S2.

Table S2. uORF characteristics

Pol Group	Virus	NCBI	Distance dVI AUG to ppAUG (nt)	uORF (nt)	UP protein (aa)	Frame vs. ppORF
I	CVA1	AF499635	126	-	*	-
	CVA19	AF499641	129	-	*	-
	CVA22	AF499643	129	-	*	-
	EV-C96	EF015886	159	195	65	+2
	EV-C113	KC344834	123	-	*	-
	EV-C116	JX514942	123	-	*	-
II	EV-C104	AB686524	132	219	73	+2
	EV-C117	JX262382	84	120	40	+2
III	EV-C105	JX514943	79	166	*	+1
	EV-C109	GQ865517	81	168	56	+1
	EV-C118	JX961708	81	168	56	+1
IV	PV1	V01149	159	195	65	+2
	PV2	M12197	159	195	*	+2
	PV3	K01392	156	192	*	+2
	CVA11	AF499636	159	195	65	+2
	CVA13	AF499637	159	195	65	+2
	CVA17	AF499639	159	195	65	+2
	CVA20	AF499642	159	204	68	+2
	CVA21	AF546702	130	-	*	-
	CVA24	D90457	159	195	65	+2
	EV-C95	KM273014	159	216	72	+2
	EV-C99	EF015008	159	195	65	+2
	EV-C102	EF555645	159	195	65	+2

* Premature termination codon (PTC)
- Not present (NP) / Not applicable (NA)

Summary of Supplemental Materials (Okolovitch et al. JVI 2025)

ClickSeq next-generation sequencing and Virus Recombination Mapper (ViReMa) were used to detect crossover sites between poliovirus and coxsackievirus A21 (CVA21), as shown in Table S1A, Table S1B and Figure S1 (1-4). Polymerases from poliovirus and CVA21 were expressed, purified and biochemically characterized (Figure S2) using methods previously described (5). Polymerase elongation complex stability assays probe functional aspects of an extended primer grip in the polymerase and its interactions with nascent RNA products (6). RNase L competitive inhibitor RNA (RNase L ciRNA) sequences from viruses in each recombination group were aligned (Figure S3). Conserved loop E motifs suggest Pol Group IV viruses contain a functional RNase L ciRNA. Viruses in Pol Groups I, II, and III contain loop E polymorphisms incompatible with functional RNase L ciRNA activity (7-9). uORFs and UP proteins are distinct from one enterovirus C polymerase group to another (Figure S4 and Table S2). uORFs begin at an AUG codon in domain VI of the IRES and overlap with the ppORF (10). uORFs and corresponding UP proteins are absent in Pol Group I viruses (but for one exception, EV-C96). uORFs are found in Pol Group II, III and IV viruses; however, the UP proteins encoded therein are distinct from one polymerase group to another. Premature termination codons (*PTC), deletions (--) and indels disrupt expression of some UP proteins. For uORF characteristics, see Table S2.

References

1. **Jaworski E, Routh A.** 2018. ClickSeq: Replacing fragmentation and enzymatic ligation with click-chemistry to prevent sequence chimeras. Methods Mol Biol 1712:71-85.

2. **Routh A, Head SR, Ordoukhanian P, Johnson JE.** 2015. ClickSeq: Fragmentation-free next-generation sequencing via click ligation of adaptors to stochastically terminated 3'-azido cDNAs. J Mol Biol 427:2610-6.

3. **Routh A, Johnson JE.** 2014. Discovery of functional genomic motifs in viruses with ViReMa-a Virus Recombination Mapper-for analysis of next-generation sequencing data. Nucleic Acids Res 42:e11.

4. **Routh AJ, Jaworski E.** 2024. ClickSeq: Randon-primed protocol with single indexing using ClickSeq Kit doi:dx.doi.org/10.17504/protocols.io.n92ld8jkov5b/v1. Springer Nature, protocols.io.

5. **Kortus MG, Kempf BJ, Haworth KG, Barton DJ, Peersen OB.** 2012. A template RNA entry channel in the fingers domain of the poliovirus polymerase. J Mol Biol 417:263-78.

6. **Kempf BJ, Watkins CL, Peersen OB, Barton DJ.** 2020. An extended primer grip of picornavirus polymerase facilitates sexual RNA replication mechanisms. J Virol 94.

7. **Han JQ, Townsend HL, Jha BK, Paranjape JM, Silverman RH, Barton DJ.** 2007. A phylogenetically conserved RNA structure in the poliovirus open reading frame inhibits the antiviral endoribonuclease RNase L. J Virol 81:5561-72.

8. T**ownsend HL, Jha BK, Han JQ, Maluf NK, Silverman RH, Barton DJ.** 2008. A viral RNA competitively inhibits the antiviral endoribonuclease domain of RNase L. RNA 14:1026-36.

9. **Townsend HL, Jha BK, Silverman RH, Barton DJ.** 2008. A putative loop E motif and an H-H kissing loop interaction are conserved and functional features in a group C enterovirus RNA that inhibits ribonuclease L. RNA Biol 5:263-72.

10. **Lulla V, Dinan AM, Hosmillo M, Chaudhry Y, Sherry L, Irigoyen N, Nayak KM, Stonehouse NJ, Zilbauer M, Goodfellow I, Firth AE.** 2019. An upstream protein-coding region in enteroviruses modulates virus infection in gut epithelial cells. Nat Microbiol 4:280-292.
